# An Alternative Approach to Compute the Likelihood for the DAISIE (Dynamic Assembly of Island Biota Through Speciation, Immigration, and Extinction) Model

**DOI:** 10.1007/s11538-026-01714-3

**Published:** 2026-08-14

**Authors:** Ornela N. Dehayem, Bart Haegeman, Rampal S. Etienne

**Affiliations:** 1 Groningen Institute for Evolutionary Life Sciences, University of Groningen, Box 11103, 9700 CC Groningen, The Netherlands; 2 Naturalis Biodiversity Center, Darwinweg 2, 2333 CR Leiden, The Netherlands; 3 Laboratory of Microbial Oceanography, CNRS/Sorbonne Université, 1 avenue Pierre Fabre, 66650 Banyuls-sur-Mer, France

**Keywords:** Macroevolution, Biodiversity patterns, Diversification model, Island biogeography, Phylogenetic trees

## Abstract

Understanding island biodiversity requires studying the processes driving its assembly and examining how these processes vary over time, across lineages, and across different islands. The DAISIE (Dynamic Assembly of Island biota through Speciation, Immigration, and Extinction) framework has been developed for this purpose, and has advanced our understanding of island community assembly by estimating, from phylogenetic data, the contribution of the processes of colonization, speciation and extinction to island community assembly. However, the model assumes uniform colonization and diversification rates across lineages, ignoring potential variation in these rates caused by, for example, lineage-specific traits. This assumption thus restricts the model’s capacity to capture complex colonization and diversification dynamics, and may consequently bias inference of colonization and diversification dynamics on islands. An extension of the framework behind these more complex dynamics is therefore desired. However, for this state-dependent colonization and diversification extension, the current computation of the likelihood of the model given phylogenetic data is computationally prohibitive. In this study, we therefore propose an alternative approach to computing the DAISIE likelihood, under the assumption of diversity-independent colonization and diversification. Our novel approach is based on the pruning algorithm that has been used for computing the likelihood of SSE (State-dependent Speciation and Extinction) models, which traces lineage history backward in time from the present day to the root of the tree. We demonstrate that our alternative approach reproduces DAISIE’s predictions under diversity-independent colonization and diversification. This provides an additional layer of support to DAISIE in diversity-independent settings, but in doing so also gives more confidence in the results under diversity-dependence. Furthermore, our alternative approach offers greater computational efficiency. Finally, it provides a flexible framework for incorporating state-dependent dynamics in colonization, speciation, and extinction processes.

## Introduction

Biodiversity, the variety of life forms on Earth, is a fundamental aspect of the natural world that underpins ecosystem function and resilience (Yadav et al. 2023; O’Connor et al. 2021). Understanding the processes that create and sustain biodiversity is a central goal in ecology and evolution. One of the primary approaches to studying the accumulation of biodiversity over evolutionary time scales is to reconstruct the past dynamics of the two evolutionary processes, speciation and extinction, that are thought to shape species richness over these time scales (Morlon et al. 2022; Morlon 2014). Diversification models fitted to time-calibrated phylogenies of extant species have been widely used to test evolutionary scenarios that may have shaped patterns of diversification through time (Nee et al. 1309; Maddison et al. 2007; Etienne et al. 2012; Etienne and Rosindell 2012; Morlon 2014; Condamine et al. 2013). Each model encodes a distinct scenario of speciation and extinction dynamics − either constant-rate, diversity-dependent, time-dependent, trait-dependent, or assuming protracted speciation. These hypotheses are evaluated by fitting a range of candidate models to identify the scenario that best approximates diversification patterns.

Marine islands present ideal systems to unravel evolutionary processes that have shaped present-day life due to their isolation, well-defined boundaries, and often exceptionally high biodiversity (Losos and Ricklefs 2009; Warren et al. 2015; Gillespie and Roderick 2014; Helmus et al. 2014; Patino et al. 2017). Their relatively recent geological origins and limited species richness compared to the much larger continental regions make studying processes shaping biodiversity more tractable. Moreover, islands (in an archipelago or across the globe) offer repeated natural experiments, providing multiple case studies for testing and contrasting evolutionary patterns across diverse environments, time and lineages (Losos and Ricklefs 2009). Island communities assemble through in situ speciation and extinction as well as via colonization (MacArthur and Wilson 2001). The DAISIE model (Dynamic Assembly of Island biota through Speciation, Immigration, and Extinction) has been developed to incorporate these three fundamental processes driving island diversity (Valente et al. 2015; Etienne et al. 2023). DAISIE makes use of information contained in phylogenies to assess the role of the processes of colonization (arrival and establishment of new island species from a nearby continent), speciation via anagenesis (evolution to an island species distinct from the mainland ancestor due to reduced gene flow between island and mainland populations) and cladogenesis (the process of an ancestral species giving rise to two different species on the island or archipelago), and extinction (loss of species from the island) in shaping island communities. It also assesses whether the rates of colonization and cladogenesis depend negatively on island diversity, and hence limit diversity.

The DAISIE framework has been successfully applied to 1) quantify how colonization, speciation, and extinction rates vary with island age, area, and isolation (Valente et al. 2020); 2) assess the role of diversity limits and equilibrium processes in shaping patterns of species richness of birds in the Galápagos and Macaronesian islands (Valente et al. 2015, 2017); 3) estimate the amount of evolutionary time lost or threatened due to anthropogenic extinctions (Valente et al. 2017, 2019); 4) demonstrate how environmental changes, such as lake expansion, can elevate equilibrium diversity by enhancing colonization rates (Hauffe et al. 2020), and 5) study the phylogenetic limits to diversity-dependence (Etienne et al. 2023).

While the DAISIE framework has been instrumental in advancing our understanding of community assembly on islands, the current DAISIE model for which parameter estimation is possible through maximum likelihood has certain limitations. The likelihood computation currently requires uniform diversification and extinction parameters across lineages, thus ignoring variability associated with species-specific traits. However, species differ in dispersal abilities, physiological and morphological traits, habitat preferences and ecological interactions, all of which can modulate colonization frequency, speciation speed, and extinction risk. Ignoring such trait dependence can bias model inferences, especially if species’ traits shape the structure of phylogenetic trees on islands (Xie et al. 2023; Maddison 2006; Maddison et al. 2007). Although DAISIE has been shown to effectively reconstruct biodiversity dynamics in many trait-dependent scenarios, it fails in some scenarios, notably when applied to phylogenetic data of community with large clade size variation (Xie et al. 2023). This limitation highlights the need for a trait-explicit modeling framework. However, extending the DAISIE framework to account for trait dependence is not straightforward, as the underlying mathematical formulation is not readily designed to incorporate trait-specific diversification and colonization dynamics. Here, we introduce an alternative methodology for modeling community assembly in isolated ecosystems under the assumption of diversity-independent diversification. Our approach follows the SSE (state-dependent speciation and extinction) framework introduced by Maddison et al. (2007), which operates backward in time, tracing lineage histories from the present day back to the island’s origin. We demonstrate that our method aligns with DAISIE’s predictions, while improving computational efficiency by making optimal use of the assumption on diversity-independence. Moreover, it provides a flexible framework that allows the incorporation of state-dependent colonization, speciation, and extinction processes. Our aim here is to present the framework rather than applying it in detail to a trait-dependent model, but we do provide an example of how our approach can be extended to incorporate trait-dependence.

## Model and Likelihood Computation

### Data Description and Assumptions

**Fig. 1:**
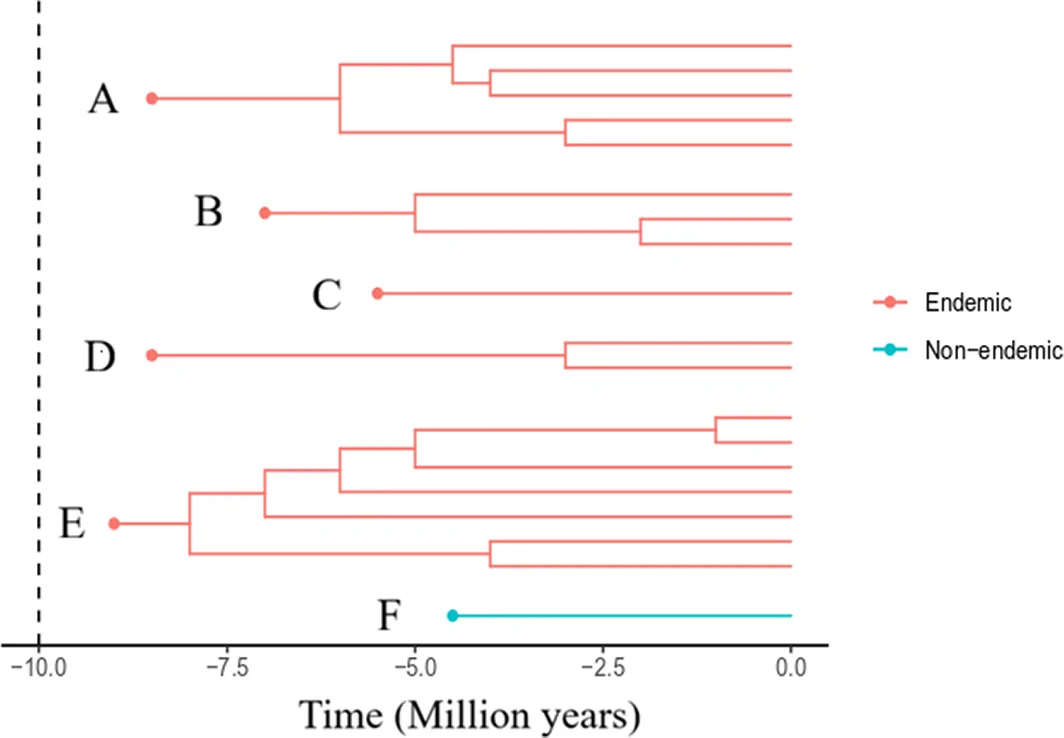
Example of a dataset in the DAISIE framework. Each tree represents a colonization event with one or more descendants on the island. Circles indicate the time of colonization of each lineage; red - endemic lineages; blue - non-endemic lineages. The clades *A*, *B*, *D*, and *E* illustrate cases where a mainland species colonizes the island and subsequently gives rise to multiple descendant species. *C* corresponds to a scenario where a mainland species colonizes the island and speciates via anagenesis or cladogenesis, but only a single species survives until the present. *F* represents a scenario where a mainland species colonizes the island and does not undergo speciation. Time runs from the island age -10 (i.e., 10 million years ago) to the present time 0

Our new methodology consists of an alternative approach to compute the likelihood of the DAISIE model as laid out in Valente et al. (2015). The DAISIE model assumes that after the emergence of an island, species can immigrate to the empty island from a mainland with a fixed species richness. Each colonization event initiates an independent lineage that may lead to 1) a single non-endemic species (same as the mainland ancestor), 2) a singleton endemic species (via anagenesis or cladogenesis followed by extinction), 3) a radiation of endemic species (via cladogenesis), or 4) extinction without leaving any extant descendants. The island community is thus represented by one or more such lineages, each corresponding to a separate colonization event and represented by its own phylogenetic tree. Figure 1 provides an example of a dataset for an island community. Each clade represents a separate colonization event from the mainland that has led to one or more species on the island. The dots represent the estimates of the colonization times, which are inferred by measuring the genetic divergence between island species and their mainland sister species (or mainland populations of the same species in the case of a non-endemic species). For the cases where these colonization times are unknown, the model integrates over all possible colonization times, that is, between the island-archipelago age and the present for singleton lineages (e.g., C and F), and between the island-archipelago age and the crown age for an endemic clade (e.g., A, B, D, E).

It is possible that before an observed colonization event, the same mainland species already colonized the island (Caujapé-Castells et al. 2017) (and persisted until the observed colonization event), but the model assumes that the observed lineage descends from the latest colonization event. This means that at the observed colonization event, any trace of these previous colonizations is erased (Valente et al. 2015). Recolonization of the same mainland ancestors can occur and still remain on the island, but only after the previous colonization has been followed by speciation. For this reason, the likelihood calculation assumes recolonization by the same mainland species is only possible after the island species undergoes speciation, because if this happened before speciation, the recolonizing population would have replaced the entire island population, and the most recent colonization event would be considered the new divergence time from the mainland which would be in disagreement with the data. Here we follow the same assumption; we do not question its validity in the current paper but will leave this for a future exploration. DAISIE can accurately estimate colonization and diversification rates within island communities in most cases from colonization times of independent lineages, their branching times, endemicity status (derived from phylogenetic data), and the geological age of the island (Neves et al. 2021; Xie et al. 2023; Lambert et al. 2022). The model also allows for diversity-dependence in colonization and cladogenetic speciation to act either within clades (clade-specific, CS model) (Valente et al. 2015) or across the entire island community (island-wide, IW model) (Etienne et al. 2023). We note that for our methodology to work we assume that, in contrast to DAISIE, there is no diversity-dependent colonization or cladogenesis. Another key difference lies in how the two models handle incomplete data sampling. DAISIE uses *n*-sampling (Etienne et al. 2012), which assumes that, given a phylogenetic (or reconstructed) tree with a known number of extant species *S*, exactly *n* species are not sampled. In contrast, our approach uses the ρ-sampling scheme (FitzJohn et al. 2009), where the phylogeny is assumed to be a random sample of all extant species, where each species is included in the phylogeny with probability ρ, and with probability 1-ρ to be unsampled. The two types of sampling are related by

1 $$\begin{aligned} P_{\rho }(\text {clade})=\sum _{n=0}^{\infty } {P_n(\text {clade}) \left( {\begin{array}{c}S + n\\ n\end{array}}\right) \rho ^S (1 - \rho ) ^{n}} \end{aligned}$$

where *S* is the number of species in the clade. In practice, ρ-sampling formulas are also applied to datasets consisting of a phylogeny with *S* tips together with a known number *m* of missing species. In that case, one typically sets $\rho = \frac{S}{S + m}$, corresponding to the observed fraction of sampled species. However, this should be seen as a pragmatic approximation rather than an exact equivalence to *n*-sampling.

### Likelihood Computation

Mainland species are considered independent of one another, allowing the dynamics of each colonizing species to be modeled separately (CS model, see above). The likelihood of observing the entire community is then calculated as the product of the probabilities of each independent colonization plus its potential descendants present on the island (which we will refer to as “lineage” hereafter even if it is a non-endemic singleton), and accounting for potential colonization events that occurred before and after the observed colonization, and subsequently went extinct. The model distinguishes four possible outcomes following a colonization event, the last three of which correspond to extant lineages observed on the island:

- No surviving lineage: a colonization event that resulted in a lineage that has gone completely extinct and is not represented among the present-day species on the island.
- Non-endemic singleton: a species present on the island that originated from a colonization event and has not undergone speciation since arrival, and is also found on the mainland.
- Endemic singleton: a species present on the island that originated from a colonization event and has undergone anagenetic or cladogenetic speciation, but only one species survives until the present on the island.
- Endemic radiation: a clade of two or more species that all descended from a single colonization event and underwent cladogenetic speciation on the island, resulting in a group of endemic species.

To compute the probabilities of observing a clade, DAISIE uses a hidden Markov formulation based on the variables $Q_n^k(t)$, which describe the probability that the diversification process is consistent with the observed phylogeny at time t, where k denotes the number of lineages represented in the phylogeny and n denotes the total number of additional (now extinct or unsampled) species present on the island at that time. The probabilities $Q_n^k(t)$ are computed forward in time, from the initial time $t_0$, corresponding to the origin of the island, to the present time $t_p$.

This formulation becomes difficult to extend to trait-dependent models because it requires tracking not only the species represented in the phylogeny, but also the species that are not represented. To illustrate this, consider a time interval during which k species are represented in the phylogeny. If the trait has m possible states, the trait states of these k represented species can occur in $m^k$ possible configurations. In addition, we must keep track of how many non-represented species occur in each of the m trait states. In principle the number of non-represented species is unbounded; however, we assume that it is bounded above by $n_{\max }$. Under this assumption, the number of possible configurations of non-represented species is $$\left( {\begin{array}{c}n_{\max } + m\\ n_{\max }\end{array}}\right) $$.

Thus, the total number of equations that must be tracked in a trait-dependent version of the current DAISIE framework scales as $$m^k \left( {\begin{array}{c}n_{\max }+m\\ n_{\max }\end{array}}\right) $$. This becomes computationally prohibitive as either the number of represented species or the number of trait states increases.

In contrast to the original DAISIE model, which traces lineage history forward in time from the island’s origin to the present, the approach we present in this paper traces it backward, from the present day to the island’s origin. Trait dependence can then be incorporated by indexing these quantities, and the associated rates, by trait state. This gives a much more compact system of equations, whose size is independent of the number of represented species and scales linearly with the number of trait states.

We start by defining the backward time ${\tau }$, measured from the present to the past. It is related to the usual forward time *t* by the equation ${\tau } = {\tau }_p - t + t_p$, where ${\tau }_p$ is the backward time at the present. We follow the differential equation-based approach introduced by Maddison et al. (2007), where probabilities are assigned at the tips of the lineage, and a set of rules is used to iteratively update these values as we trace the lineage backward in time, from the tips to the colonization time. At the colonization time of a lineage, we incorporate the probability of colonization, before continuing the computation further back in time to the island’s origin.

**Fig. 2:**
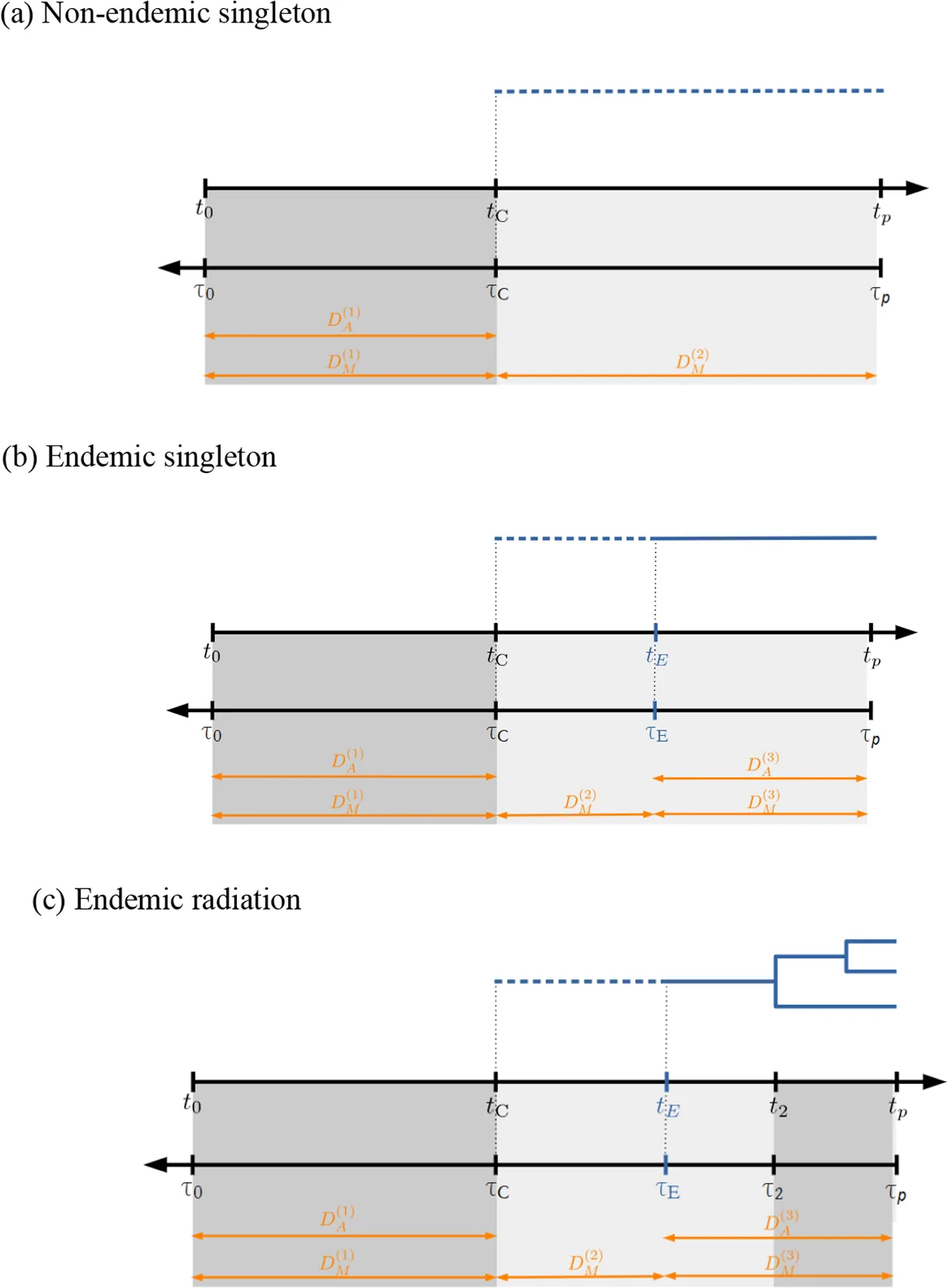
Schematic representations of the temporal structure and definition of the state probabilities ${D}_{\textrm{M}}({{\tau }})$ and $D_{\textrm{A}}({{\tau }})$ in the backward pruning algorithm for different cases. The dashed blue line represents the time interval during which the species is non-endemic. The blue solid line represents the time interval during which the species is endemic. The orange arrows show the time intervals over which each probability is computed, going backward in time. The grey-shaded regions represent different time intervals, each governed by a distinct set of differential equations. These intervals correspond to different phases in the lineage history, reflecting changes in model dynamics across time. The times shown in black (${\tau }_0$, ${\tau }_C$, ${\tau }_2$, and ${\tau }_p$) are extracted from the data. In contrast, the time points shown in blue (${\tau }_E$) are realization-dependent. These times are described as follows: ∙ $t_0, {\tau }_0$: time of the island’s origin, i.e. the island’s age; ∙ $t_C, {\tau }_C$: time of colonization event that gives rise to lineage observed at the present time; ∙ $t_E, {\tau }_E$: time at which the endemic lineage at the present time becomes endemic; ∙ $t_p, {\tau }_p$: present time

We are now ready to define two central probabilities used in the model:

- $E({{\tau }})$, the probability that a lineage starting at time ${\tau }$ as an endemic species leaves no descendants at the present;
- $D({{\tau }})$, the probability that a lineage alive at time ${\tau }$ leaves a clade that is consistent with the observed data. In order to capture the state of the system at specific points in time, we introduce several state-dependent variants of the probability $D({{\tau }})$. These states represent the probabilities of the dynamics being consistent with the observed data. We define these states as follows:
  - ${D}_{\textrm{E}}({{\tau }})$ is the probability that a lineage that started on the island at time ${\tau }$ as an endemic lineage evolves into the observed clade;
  - ${D}_{\textrm{M}}({{\tau }})$ is the probability that all lineages descending from the mainland species present at time ${\tau }$, as well as those originating from subsequent colonizations, evolve in a manner consistent with the observed data;
  - $D_{\textrm{A}}({{\tau }})$ is the probability that the mainland species is absent from the island at time ${\tau }$, and that all lineages resulting from subsequent colonizations evolve in a manner consistent with the observed data.
  Fig. 2 provides a schematic representation of the state probabilities $D_{\textrm{M}}({{\tau }})$ and $D_{\textrm{A}}({{\tau }})$ defined in the model.

We construct the likelihoods by integrating a series of differential equations. To formulate these equations, it is convenient to introduce different instances of the variables $D_{\textrm{A}}$ and ${D}_{\textrm{M}}$:

- ${D}_{\textrm{M}}^{(1)}({{\tau }})$, defined for ${\tau } \in [{\tau }_C, {\tau }_{0}]$, is the probability that the dynamics result in a clade consistent with the data, starting at time ${\tau }$ from an island with the non-endemic species which is not represented in the clade. This mainland species originates from a colonization event between ${\tau }_{0}$ and ${\tau }_{C}$. In this state the mainland species goes extinct before the present, or is taken over by the observed mainland species;
- ${D}_{\textrm{M}}^{(2)}({{\tau }})$, defined for ${\tau } \in [{\tau }_{p}, {\tau }_C]$, is the probability that the dynamics result in a clade consistent with the data, starting at time ${\tau }$ from an island with the non-endemic species which is represented in the clade. This mainland species originates from a colonization at ${\tau }_{C}$. In this state the mainland species can speciate to initiate the observed lineage;
- ${D}_{\textrm{M}}^{(3)}({{\tau }})$, defined for ${\tau } \in [{\tau }_p, {\tau }_E]$, is the probability that the dynamics result in a clade consistent with the data, starting at time ${\tau }$ from an island with the non-endemic species which is not represented in the clade. This mainland species originates from a colonization after the speciation event on the observed lineage at ${\tau }_E$. In this state the mainland species goes extinct before the present;
- ${D}_{\textrm{A}}^{(1)}({{\tau }})$, defined for ${\tau } \in [{\tau }_C, {\tau }_{0}]$, is the probability that the mainland species $D_{\textrm{M}}^{(1)}$ is absent from the island at time ${\tau }$;
- ${D}_\mathrm{{A}}^{(3)}({{\tau }})$, defined for ${\tau } \in [{\tau }_p, {\tau }_{E}]$, is the probability that the mainland species $D_{\textrm{M}}^{(3)}$ is absent from the island at time ${\tau }$.

Let us consider a lineage present on an island, with estimated colonization time ${\tau }_{C}$, which may or may not have diversified into extant species. At the time ${\tau }_{C}$ the mainland ancestor colonized the island at a colonization rate γ. Following colonization, the lineage can diversify via cladogenesis at a rate $\lambda ^c$, or anagenesis at a rate $\lambda ^a$, or may go extinct at a rate μ. The likelihood of each clade is given by $D_{\textrm{A}}^{(1)}( {\tau }_0)$, which represent the probability that a process starting from an empty island at the time of island emergence ${\tau }_0$ gives rise to the observed lineage on the island at time ${\tau }_p$.

We now describe how to compute the likelihood for two specific cases: 1. no surviving species on the island and 2. a lineage with two or more species, assuming that the colonization time ${\tau }_C$ is known. The equations for singleton lineages can be derived analogously and are provided in Appendix A. The same framework can also be extended to the case where the colonization time is not precisely known, but is constrained to lie between ${\tau }_{\min }$ and ${\tau }_{\max }$. These extensions are also given in the Appendix A.

#### No Surviving Descendants

In this scenario, mainland species may have colonized, but none of the colonists survive to the present ${\tau }_{p}$. The dynamics of the mainland species on the island are captured by the pair of variables ${D_{\textrm{M}}^{(1)}}$ and $D_{\textrm{A}}^{(1)}$. These state probabilities depend on the time ${\tau }$ (see Table 3). But for notational simplicity we omit this dependence in all equations throughout the manuscript. The dynamical equations describing the change in the probabilities from the present time ${\tau }_p$ to time of the island’s origin ${\tau }_0$ are given by:

**Figure:**
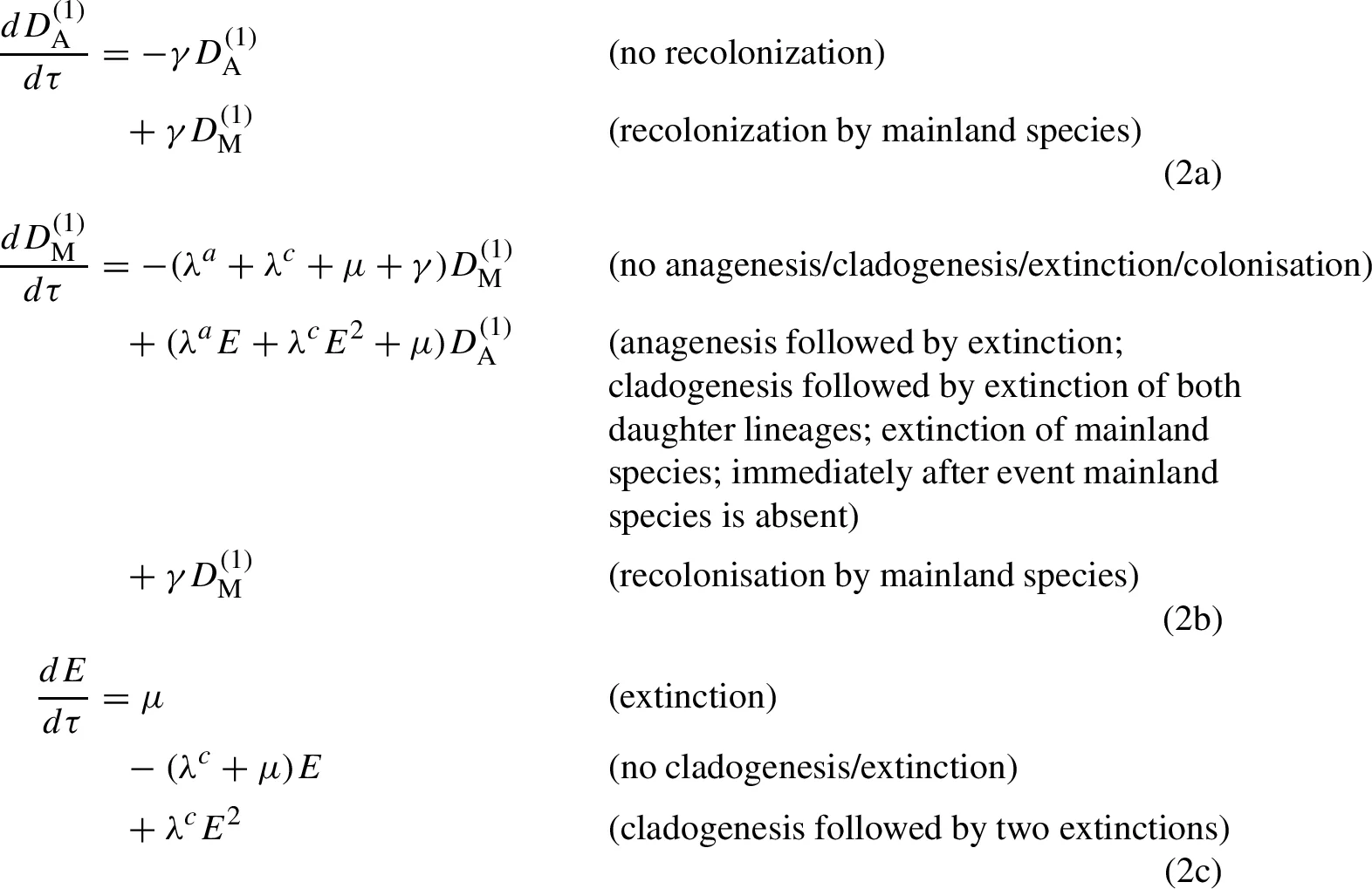

In the equation for $D_{\textrm{M}}^{(1)}$, the term $+\gamma D_{\textrm{M}}^{(1)}$ cancels with the recolonisation component $-\gamma D_{\textrm{M}}^{(1)}$ contained in $-(\lambda ^a+\lambda ^c+\mu +\gamma )D_{\textrm{M}}^{(1)}$.

These equations describe the scenario in which lineages colonize the island at some point between ${\tau }_p$ and ${\tau }_0$, may or may not undergo speciation through anagenesis or cladogenesis, but all resulting lineages eventually go extinct before the present.

At the present time ${\tau }_p$, the initial conditions are:

3 $$\begin{aligned} D^{(1)}_{\textrm{A}}({\tau }_p) = 1,\quad D^{(1)}_{\textrm{M}}({\tau }_p) = 0,\quad E({\tau }_p) = 0. \end{aligned}$$

This initialization reflects the fact that the mainland species is not observed on the island at the present time. Integrating these quantities backward to ${\tau }_0$ gives the likelihood

4 $$\begin{aligned} \mathcal {L}_{0} = D_{\textrm{A}}^{(1)}({\tau }_0), \end{aligned}$$

#### Endemic Non-singleton Lineage, with Colonization Time at ${\tau }_C$

We consider an endemic non-singleton lineage whose mainland ancestor colonises the island at time ${\tau }_C$. After colonisation, the lineage diversifies on the island and gives rise to several extant descendant species observed at the present time ${\tau }_p$. The crown age of the endemic clade is denoted by ${\tau }_2$. After the first speciation of the lineage, the mainland ancestor may recolonize the island and go extinct again; this is captured by the variables $D_\textrm{M}^{(3)}$ and $D_\textrm{A}^{(3)}$.

The likelihood is computed by integrating backward in time from the present ${\tau }_p$ towards the island age ${\tau }_0$. We divide the history of the lineage into three intervals:

$$ [{\tau }_p,{\tau }_2], \qquad [{\tau }_2,{\tau }_C], \qquad [{\tau }_C,{\tau }_0]. $$

**Dynamics between** $$[{\tau }_p,{\tau }_2]$$

Between the crown age ${\tau }_2$ and the present ${\tau }_p$, the lineage is present, and has already undergone cladogenesis speciation. The dynamical equations describing the change in the probabilities between ${\tau }_p$ and ${\tau }_2$ are given by:

**Figure:**
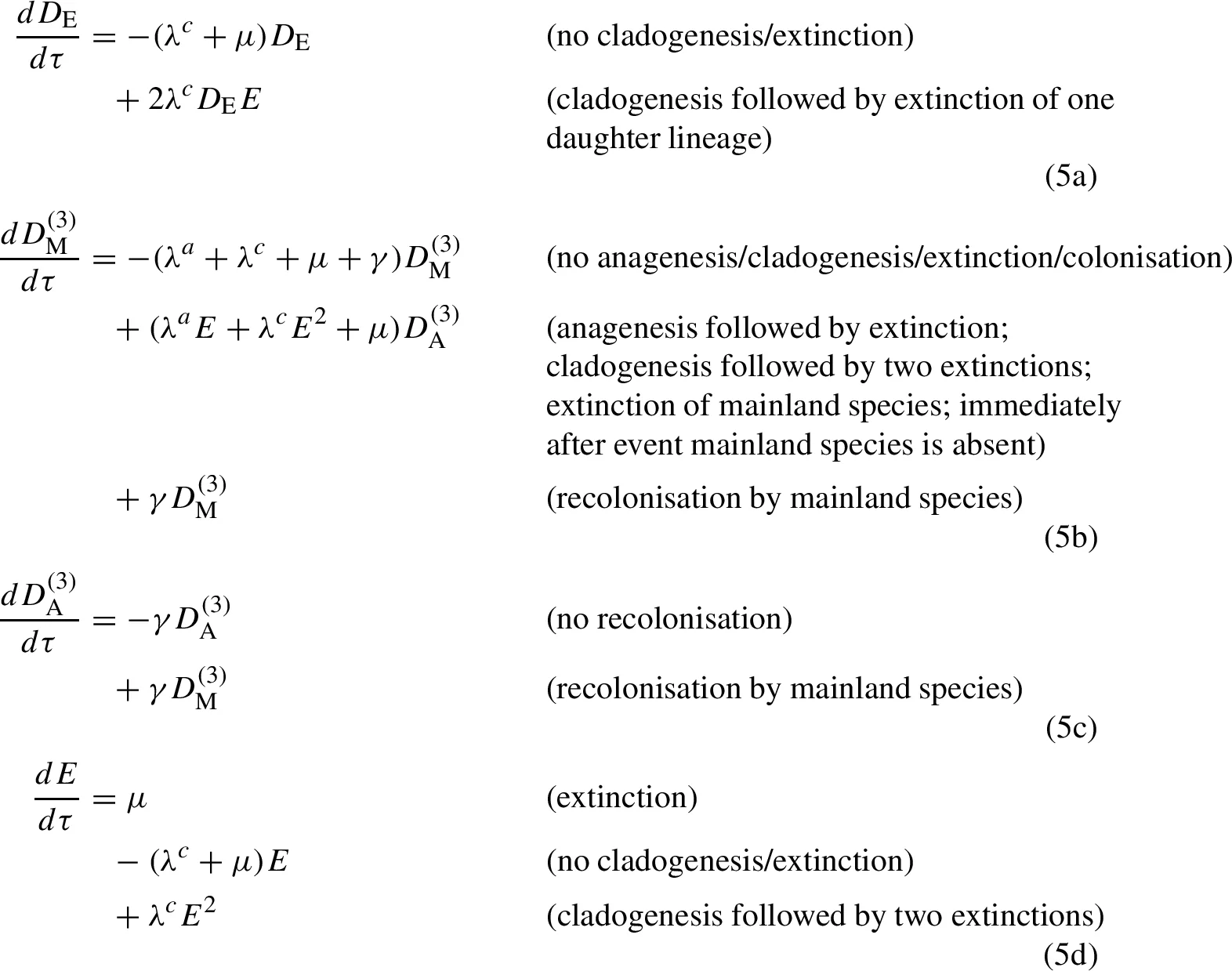

In the equation for $D_{\textrm{M}}^{(3)}$, the term $+\gamma D_{\textrm{M}}^{(3)}$ cancels with the recolonisation component $-\gamma D_{\textrm{M}}^{(3)}$ contained in $-(\lambda ^a+\lambda ^c+\mu +\gamma )D_{\textrm{M}}^{(3)}$.

The initial conditions at ${\tau }_p$ are given by:

6 $$\begin{aligned} D_\textrm{E}({\tau }_p) = 1, \quad D_\textrm{M}^{(3)}({\tau }_p) = 0, \quad D_\textrm{A}^{(3)}({\tau }_p) = 1, \quad {E}({\tau }_p) = 0 \end{aligned}$$

If species in the lineages are sampled at random (all species in the clade have the same probability of being sampled), incomplete sampling can be accounted for by modifying the initial conditions for each tip in the tree to reflect ρ, the probability of sampling a species, following FitzJohn et al. (2009). The initial conditions at ${\tau }_p$ are modified to:

7 $$\begin{aligned} D_\textrm{E}({\tau }_p) = \rho , \quad D_\textrm{M}^{(3)}({\tau }_p) = 0, \quad D_\textrm{A}^{(3)}({\tau }_p) = 1, \quad {E}({\tau }_p) = 1 - \rho \end{aligned}$$

where ρ is the sampling fraction of the lineage.

The set of equations Eqs. (5a-5d) are integrated along the edges of the trees starting from the tips. At each branching time, the probabilities of the two daughter branches are combined by multiplying both probabilities with one another and with $\lambda ^c$ (from $\lambda ^c \, \textrm{d}{\tau }$ which represents the probability of cladogenesis speciation over the small time interval $d{\tau }$). The resulting product serves as the initial condition for the ancestral (subtending) branch as we continue tracing the lineage backward toward its colonisation time.

**Dynamics between** $$[{\tau }_2,{\tau }_C]$$

In the time interval $[{\tau }_2,{\tau }_C]$, the species has already colonized and may or may not have speciated. We have to add the dynamical equation for $D_\textrm{M}^{(2)}$, because we know that on at least a part of the branch the species is non-endemic. The dynamical equations describing the change in the probabilities between ${\tau }_2$ and ${\tau }_C$ are given by:

**Figure:**
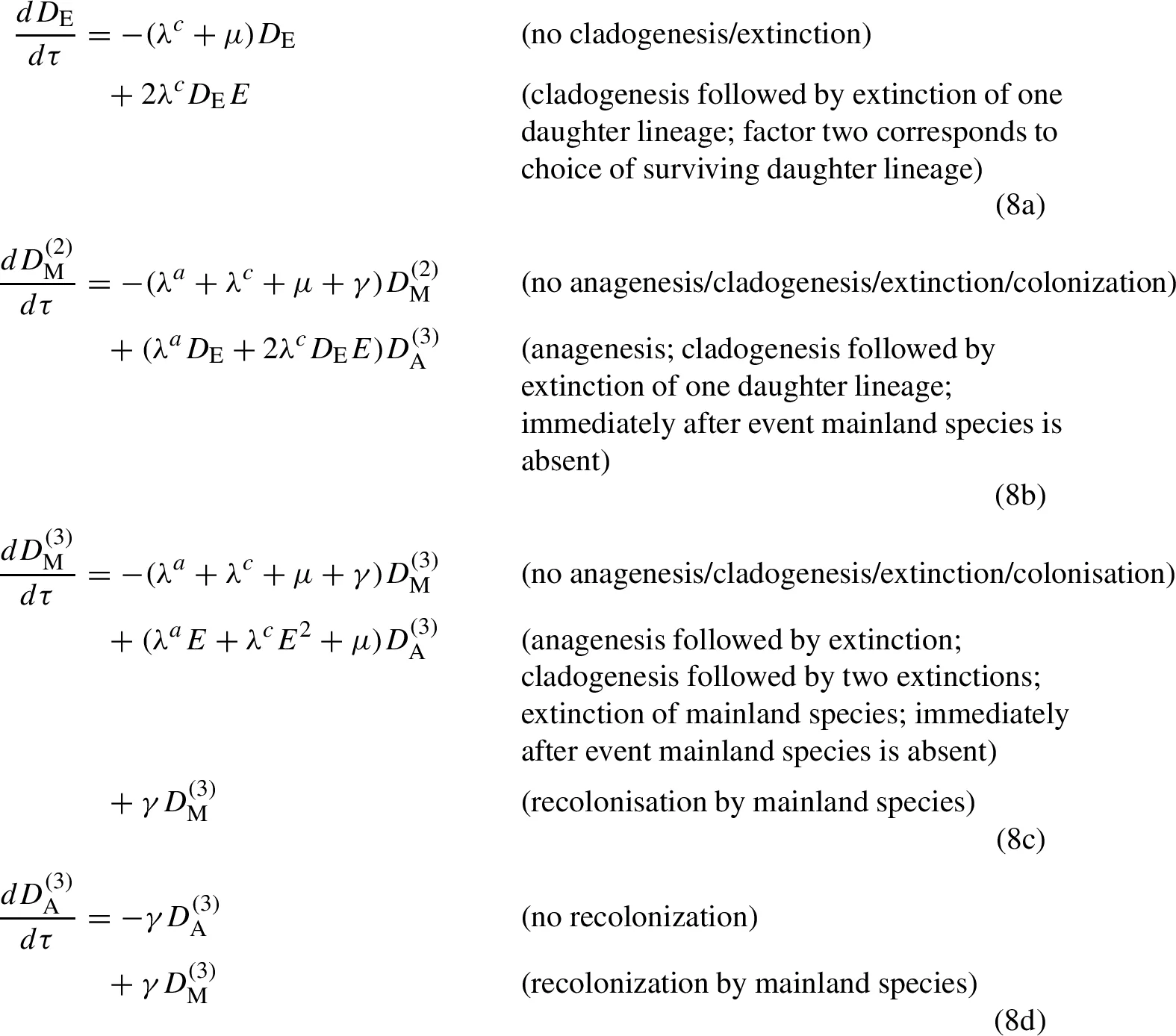

The equation is *E* are given by Eq. (2c)

As initial conditions for $D_\textrm{E}({\tau }_2^+)$ at ${\tau }_2$, we take $\lambda ^c$ times the product of the $D_\textrm{E}$ functions of the merging subclades. For $D_\textrm{M}^{(2)}({\tau }_2^+)$ we use the same quantity multiplied by the probability $D_\textrm{A}^{(3)}({\tau }_2^-)$ that the mainland species is not present on the island. This covers both cases in which the speciation event that transforms the mainland species into an endemic one occurs either between ${\tau }_C$ and ${\tau }_2$ or precisely at time ${\tau }_2$. The initial conditions at ${\tau }_2$ are thus:

9a $$\begin{aligned} D_\textrm{E} ({\tau }_2^+)= &  \lambda ^c\,\, D_{E}^{(l)}({\tau }_2^-) D_{E}^{(r)} ({\tau }_2^-) \end{aligned}$$

9b $$\begin{aligned} D_\textrm{M}^{(2)}({\tau }_2^+)= &  \lambda ^c\,\, D_{E}^{(l)}({\tau }_2^-) D_{E}^{(r)}({\tau }_2^-)D_\textrm{A}^{(3)} ({\tau }_2^-),\end{aligned}$$

9c $$\begin{aligned} D_\textrm{M}^{(3)}({\tau }_2^+)= &  D_\textrm{M}^{(3)}({\tau }_2^-),\end{aligned}$$

9d $$\begin{aligned} D_\textrm{A}^{(3)} ({\tau }_2^+)= &  D_\textrm{A}^{(3)} ({\tau }_2^-),\end{aligned}$$

9e $$\begin{aligned} E ({\tau }_2^+)= &  E ({\tau }_2^-) \end{aligned}$$

Here $D_{E}^{(l)}({\tau }_2^-)$ and $D_{E}^{(r)}({\tau }_2^-)$ are the probabilities that the two descendant lineages (left and right lineage in the phylogeny) are endemic just before ${\tau }_2$.

**Dynamics between** $$[{\tau }_C,{\tau }_0]$$

In the interval $[{\tau }_C,{\tau }_0]$ there is no clade, and so the mainland species is not represented. Still it could have colonized the island, a possibility we describe with the variable $D_{\textrm{M}}^{(1)}$. The dynamical equations describing the change in the probabilities between ${\tau }_C$ and ${\tau }_0$ are given by Eqs. (2a-2c). The initial conditions at ${\tau }_C$ are given by:

10 $$\begin{aligned} D_\textrm{A}^{(1)}({\tau }_C^+) \;=\; D_\textrm{M}^{(1)}({\tau }_C^+) \;=\; \gamma \,\,D_\textrm{M}^{(2)}({\tau }_C^-), \qquad {E}({\tau }_C^+) \;=\; {E}({\tau }_C^-). \end{aligned}$$

Integrating these quantities backward to ${\tau }_0$ gives the likelihood:

11 $$\begin{aligned} \mathcal {L}_{E} = D_{\textrm{A}}^{(1)}({\tau }_0), \end{aligned}$$

Once the likelihood is written in terms of the backward variables D and E, extending the model from a non-trait-dependent to a trait-dependent setting requires indexing the rates and probabilities by the trait state. Thus, the constant rates $\lambda ^a$, $\lambda ^c$, μ, and γ are replaced by state-dependent rates, and additional transition terms $q_{ij}$ are added to account for changes from trait state i to trait state j. To illustrate this extension, we present the trait-dependent analogue of the simplest case, i.e. the one without surviving descendants. Specifically, we consider a mainland species with trait state $i_{\textrm{M}}$ has not colonized the island, or it has, but after establishment and potential speciation or trait change, the species or any of its descendants did not survive to the present time ${\tau }_{\textrm{p}}$. The dynamical equations describing the change in the probabilities between the present time ${\tau }_p$ and the island age ${\tau }_0$ are given by:

**Figure:**
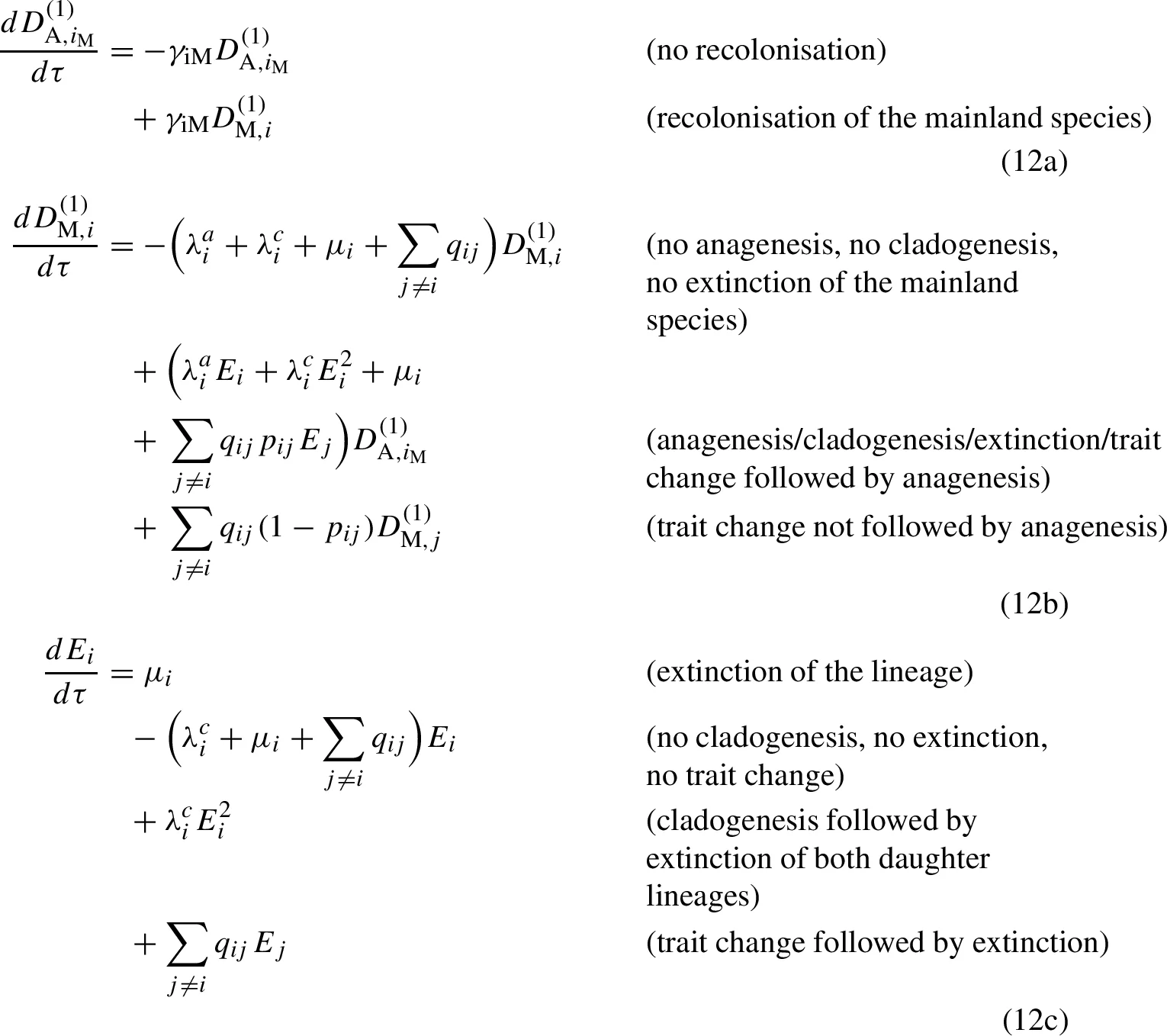

where iM represents the trait state of the mainland ancestor, the term $$\sum _{\begin{array}{c} j \ne i \end{array}} q_{ij}$$ the sum of all possible transition rates from state i to any other state j, with j≠i and $p_{ij}$ represents the probability that a change from trait *i* to *j* causes anagenesis.

At the present time ${\tau }_p$, the initial conditions are given by:

13 $$\begin{aligned} D^{(1)}_{\textrm{A},i_{\textrm{M}}}({\tau }_p) = 1,\quad D^{(1)}_{\textrm{M},i}({\tau }_p) = 0,\quad E_i({\tau }_p) = 0. \end{aligned}$$

The likelihood is given by:

14 $$\begin{aligned} \mathcal {L}_{0} = D_{\textrm{A},i_{\textrm{M}}}^{(1)}({\tau }_0), \end{aligned}$$

A complete and detailed derivation of the full trait-dependent extension of DAISIE-DE for all other scenarios will be presented in a separate paper.

We implemented these likelihood computations, including those for unknown colonization time in the R package DAISIE available on Github at https://github.com/rsetienne/DAISIE/tree/master/R, where we refer to them as DAISIE-DE, after the main equations for *D* and *E*.

## Parameter Estimates by DAISIE vs DAISIE-DE

To evaluate the performance of our new approach DAISIE-DE and, as an important independent check of DAISIE, we compared its parameter estimates with those obtained from the DAISIE model across four empirical island biota datasets (Table 1) available in the package DAISIE. For each dataset, both models were fitted to estimate the rates of colonization, cladogenesis, anagenesis, and extinction. The carrying capacity was set to K=∞ in DAISIE to eliminate the influence of diversity-dependent dynamics, allowing a comparison of the diversity-independent DAISIE and DAISIE-DE. Table 1 summarizes the resulting rate estimates. The comparison between the DAISIE and DAISIE-DE results shows that, for each dataset, the maximum likelihood and corresponding parameter estimates of the observed phylogenetic data are practically identical. The minute differences are due to numerical precision limits in integration and optimization.

It is important to note that all datasets used in this comparison were complete. When there are missing species, we cannot directly compare the likelihood we obtain from DAISIE-DE to that of DAISIE, because DAISIE uses a different kind of sampling (*n*-sampling where the number of unsampled species is specified, versus ρ-sampling which we use here, where the fraction of sampled species is specified). However, we show in Appendix A that we can compute the likelihood involving *n*-sampling from our approach involving ρ-sampling, and we demonstrate with simulations that the results are practically identical to those from DAISIE and somewhat closer to the true values than with ρ-sampling (see Figures  5-7).

**Fig. 3:**
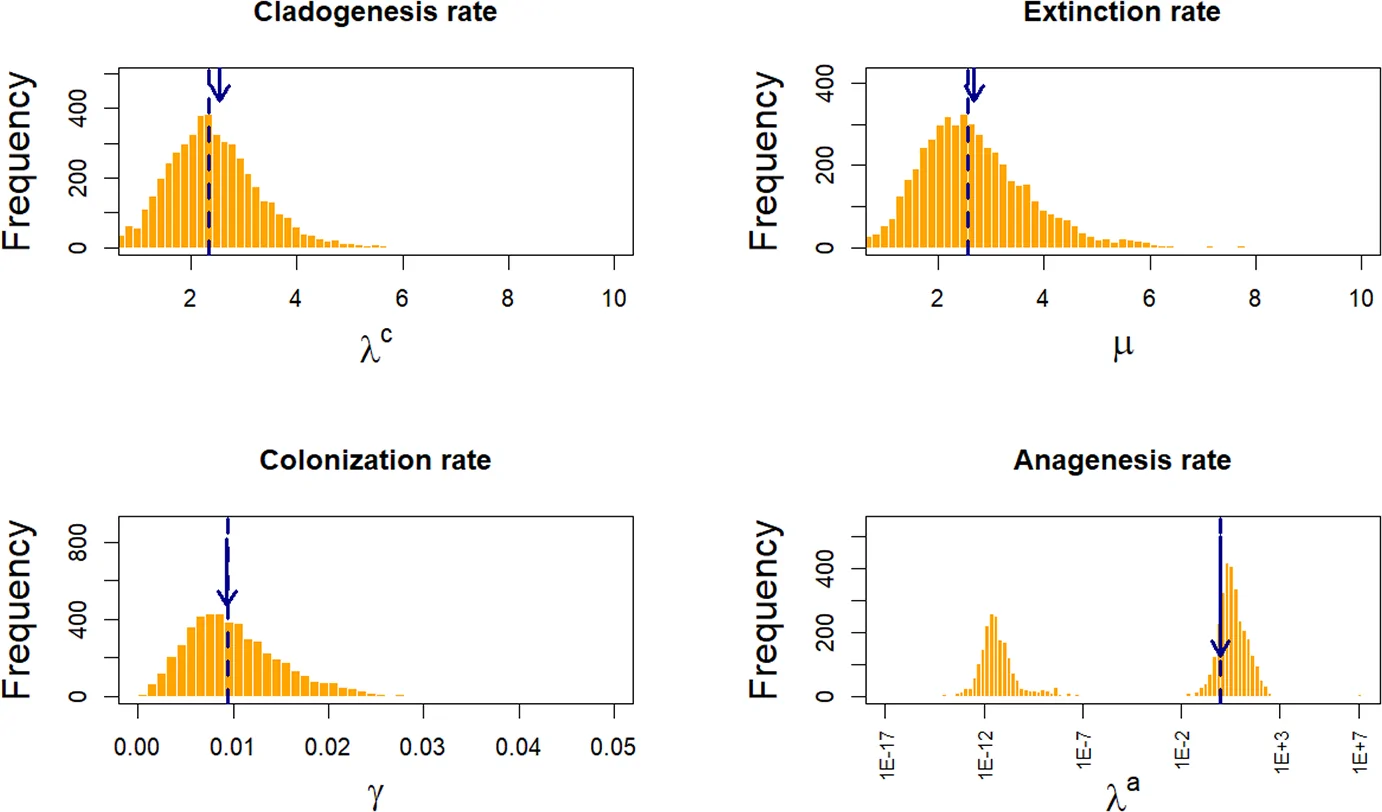
Results of the parametric bootstrap analysis obtained by fitting the model to 5000 datasets simulated with the ML parameters of one of the 4 empirical datasets (the Galapagos phylogenetic dataset). Blue arrows indicate the true parameter values used in the simulations. The anagenesis rates are plotted on a logarithmic scale, and the distribution appears to be bimodal

**Fig. 5:**
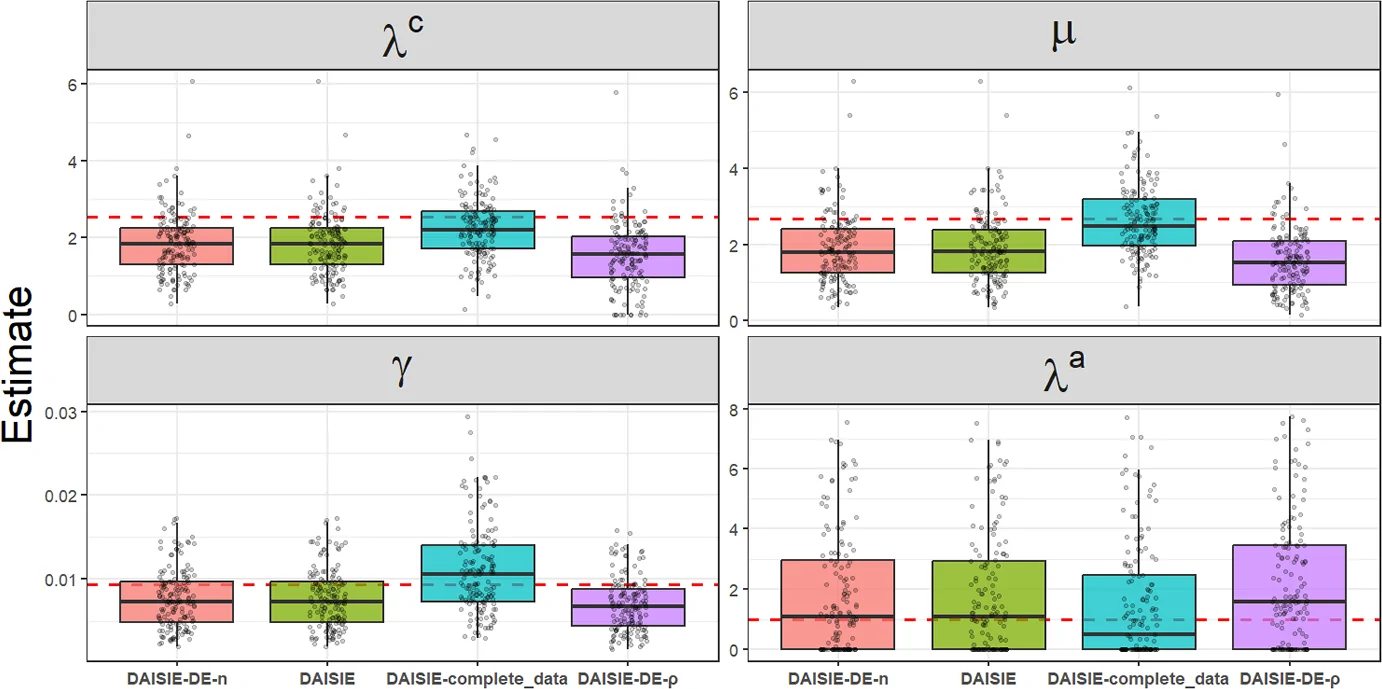
Distribution of parameter estimates across models and 100 complete and incomplete replicates. Boxplots show the variability of the maximum-likelihood estimates for each parameter across simulation replicates and fitted models. The red dashed horizontal line indicates the true parameter value used to generate the simulated data. Points correspond to individual replicates. The complete-data model is shown as a reference, while the other methods are fitted to incomplete datasets with 50% data coverage

**Fig. 6:**
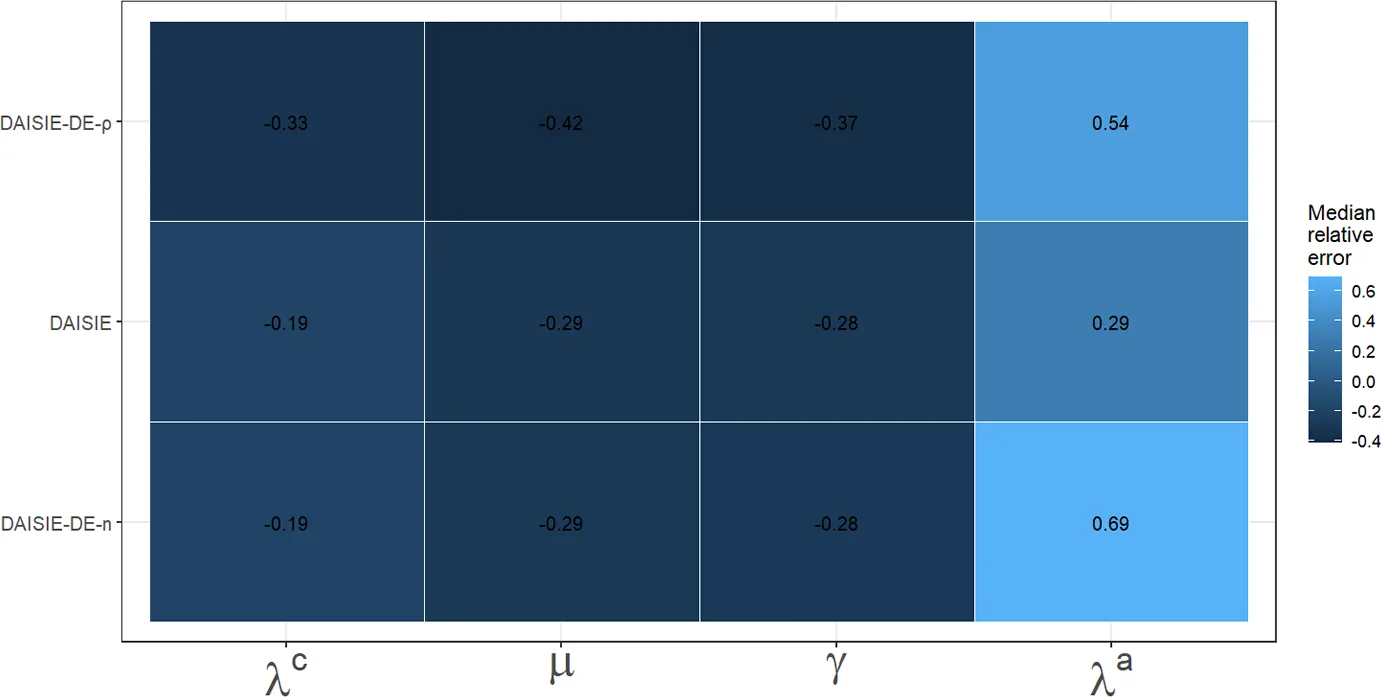
Median relative deviation of parameter estimates from the complete-data reference model. Each cell shows the median relative error of a given method compared with the estimates obtained by fitting the model to the incomplete simulation replicates with 50% data coverage. Rows correspond to the models fitted to incomplete data, and columns correspond to the four estimated parameters $\lambda ^c-$cladogenesis, μ-extinction, γ-colonization, $\lambda ^a-$anagenesis rates (color figure online)

**Fig. 7:**
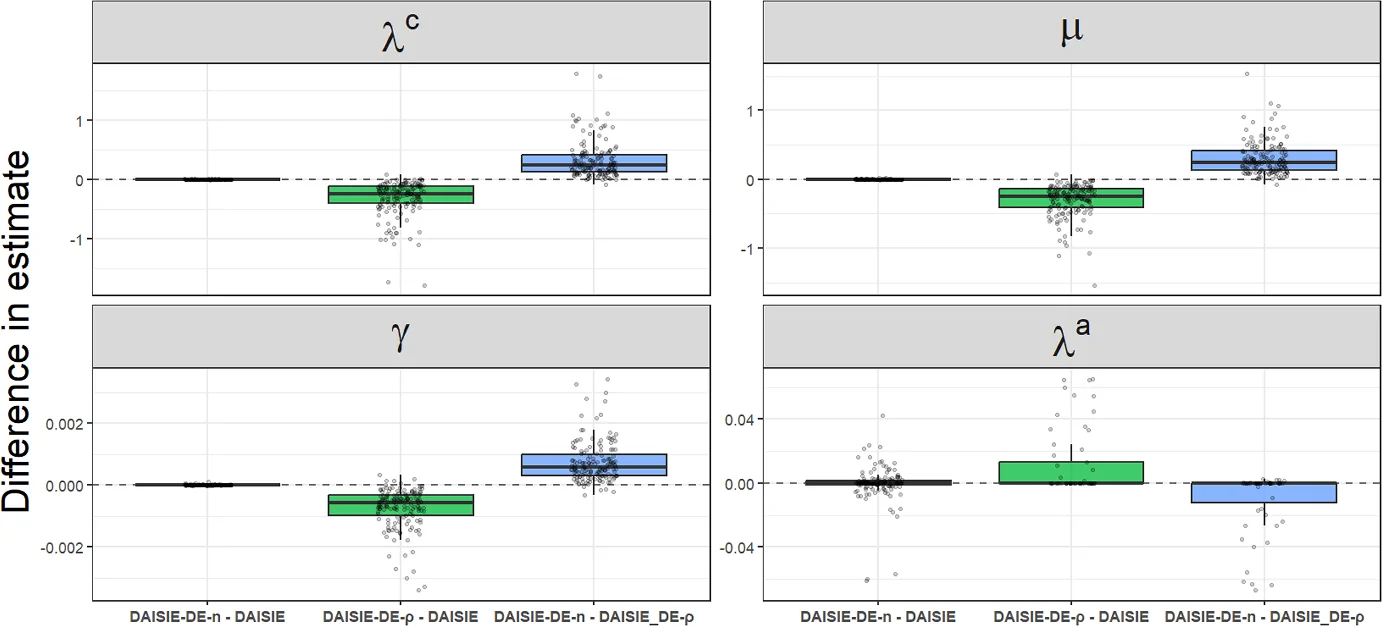
Pairwise differences between parameter estimates obtained by fitting models to incomplete data with 50% data coverage. For each parameter, boxplots show the distribution of the difference between estimates obtained with pairs of models across incomplete replicates. Deviations from zero indicate differences induced by the choice of missing-data correction (color figure online)

To further assess the robustness of DAISIE-DE’s inference, we performed a parametric bootstrap analysis using one of the four empirical datasets, the Galapagos birds dataset. We fitted the model to the Galapagos birds dataset, and we simulated 5000 datasets under the model using the ML estimated parameters from the Galapagos birds dataset. We then re-estimated parameters for each simulated dataset and compared how much these estimates vary across the 5000 replicates. Figure 3 shows the distribution of parameter estimates across the 5000 replicates. This figure shows that the median values of the estimated rates of cladogenesis, colonization, anagenesis and extinction are close to the true generating values. However, the error and variance for anagenesis are noticeably larger than for other parameters. Estimating anagenesis was already challenging under the original DAISIE model (Valente et al. 2018, 2015). This is because anagenesis is primarily informed by endemic singleton lineages, but endemic singletons can also be produced by cladogenetic diversification followed by extinction.

**Table 1:** Comparison of rates estimated by DAISIE and DAISIE-DE across different empirical datasets

Empirical	S/C	Method	Time in s	$\lambda ^c$	μ	γ	$\lambda ^a$	logLik
dataset								
Galápagos birds	25/8	DAISIE	45	2.5504	2.6835	0.00934	1.0073	−75.99
		DAISIE-DE	18	2.5506	2.6838	0.00934	1.0072	−75.99
(Valente et al. 2015)								
Hispaniola frogs	65/5	DAISIE	38	0.1787	0.0247	0.00062	0.000	−209.85
		DAISIE-DE	16	0.1787	0.0246	0.00062	0.000	−209.85
(Etienne et al. 2023)								
Cape Verde birds	10/10	DAISIE	196	0.0000	0.7678	0.0262	0.4628	−62.63
		DAISIE-DE	16	0.0000	0.7676	0.0262	0.4628	−62.63
(Valente et al. 2019)								
Biwa fish	71/68	DAISIE	1786	0.0801	1.4218	0.3917	0.3131	−230.54
		DAISIE-DE	26	0.0801	1.4218	0.3917	0.3130	−230.54
(Hauffe et al. 2020)								

The column “S/C” indicates the total number of species and colonisation events, respectively. The four empirical datasets are available in the package DAISIE

## Discussion

In this paper, we introduced an alternative approach for computing the likelihood of island-community assembly through colonisation, speciation, and extinction. The main motivation behind this reformulation was to facilitate the incorporation of additional biological complexity, such as trait dependence in colonisation and diversification rates. Such extensions are difficult to implement within the standard DAISIE likelihood framework, which is based on the Q-approach.

Table 2 compares the number of equations required under the trait-dependent extension of DAISIE, using the Q-approach, and under the trait-dependent DAISIE-DE formulation, using the backward approach.

**Table 2:** Comparison of the approximate number of equations required by a trait-dependent extension of DAISIE, using the Q-approach, and the trait-dependent extension of the DAISIE-DE formulation, presented in this paper. Here, m is the number of trait states, k is the number of species represented in the phylogeny during the time interval considered, and $n_{\max }=30$ is the maximal number of non-represented species that can be accounted for in the Q-approach, that is, it is the length of the Q-vector. For the Q-approach, the number of equations scales as $$m^k \left( {\begin{array}{c}n_{\max }+m\\ n_{\max }\end{array}}\right) $$. For the DAISIE-DE approach, we count the largest time segment as containing approximately 9m+3 trait-indexed equations

Number of trait states m	Represented species k	Q-approach equations	DAISIE-DE equations
2	5	$1.6 \times 10^{4}$	21
2	10	$5.1 \times 10^{5}$	21
2	20	$5.2 \times 10^{8}$	21
4	5	$4.7 \times 10^{7}$	39
4	10	$4.9 \times 10^{10}$	39
4	20	$5.1 \times 10^{16}$	39

To evaluate the performance of our new approach DAISIE-DE, we applied it to four empirical island biota datasets and compared its estimates of colonization, speciation (cladogenesis, anagenesis), and extinction rates with those obtained using the original DAISIE model (see Table 1). We found a strong agreement between the two models across all datasets, indicating that DAISIE-DE preserves the inferential robustness of DAISIE. In addition, DAISIE-DE is also computationally more efficient. The new likelihood calculation requires less computational time than the original DAISIE implementation. This is particularly important as the new diversity-independent model can be applied to much larger trees, making DAISIE-DE a practical alternative in these cases. Of course, one has to bear in mind that it cannot be used when colonization or diversification are diversity-dependent. More importantly, DAISIE-DE is built upon the same framework as State-dependent Speciation and Extinction (SSE) models, which opens the door to future extensions, such as incorporating trait-dependent diversification and colonization. In such a trait-dependent extension of the model, colonization, speciation, and extinction rates, as well as the probabilities associated with lineage states, would depend explicitly on species trait states, which themselves may change over time. We will explore this extension in future work. Furthermore, the similarity between DAISIE and DAISIE-DE lends support to the validity of the original DAISIE model, itself as two different approaches yield identical results across diverse empirical datasets.

## Figures and Tables

**Fig. 4:**
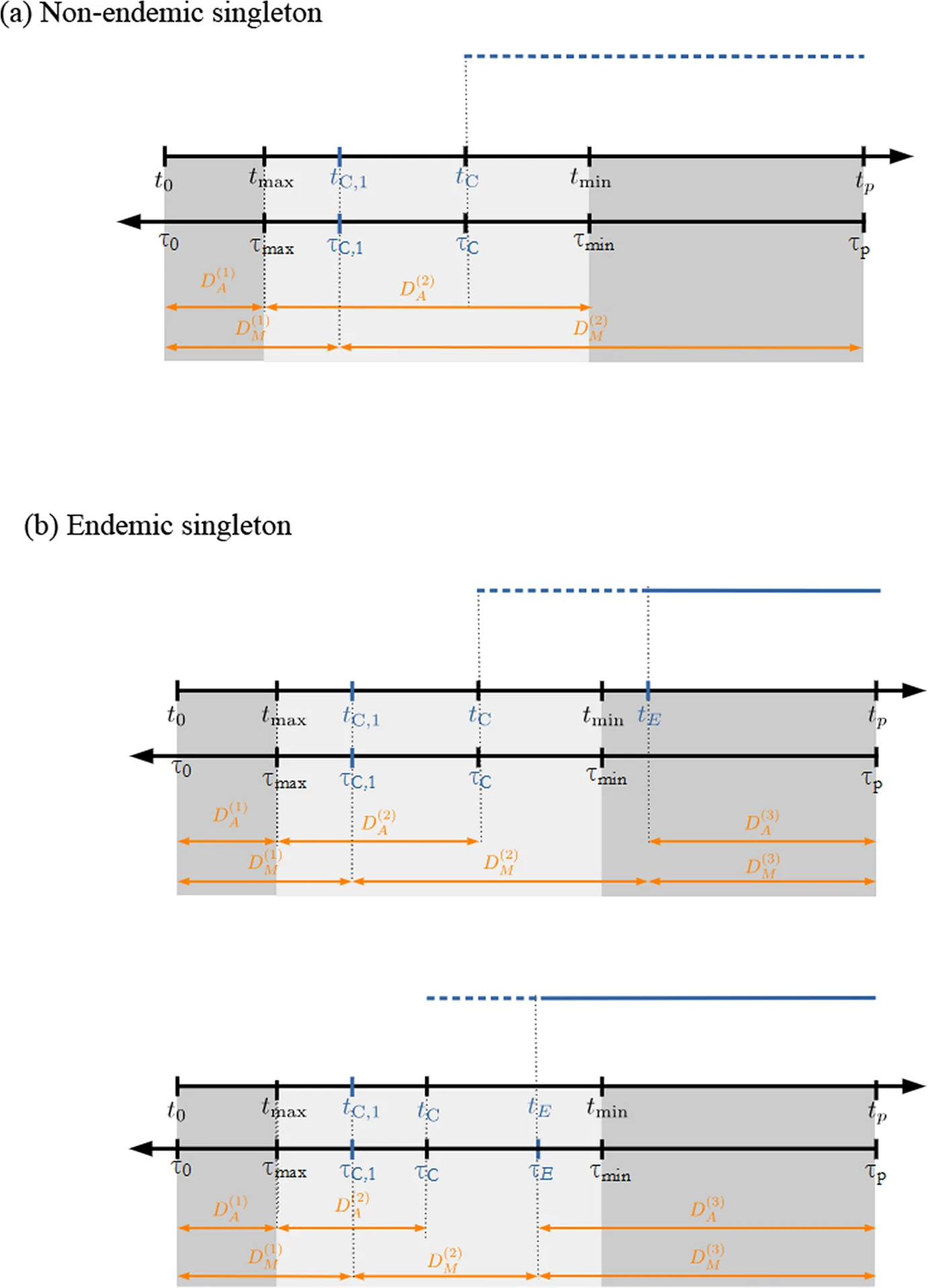

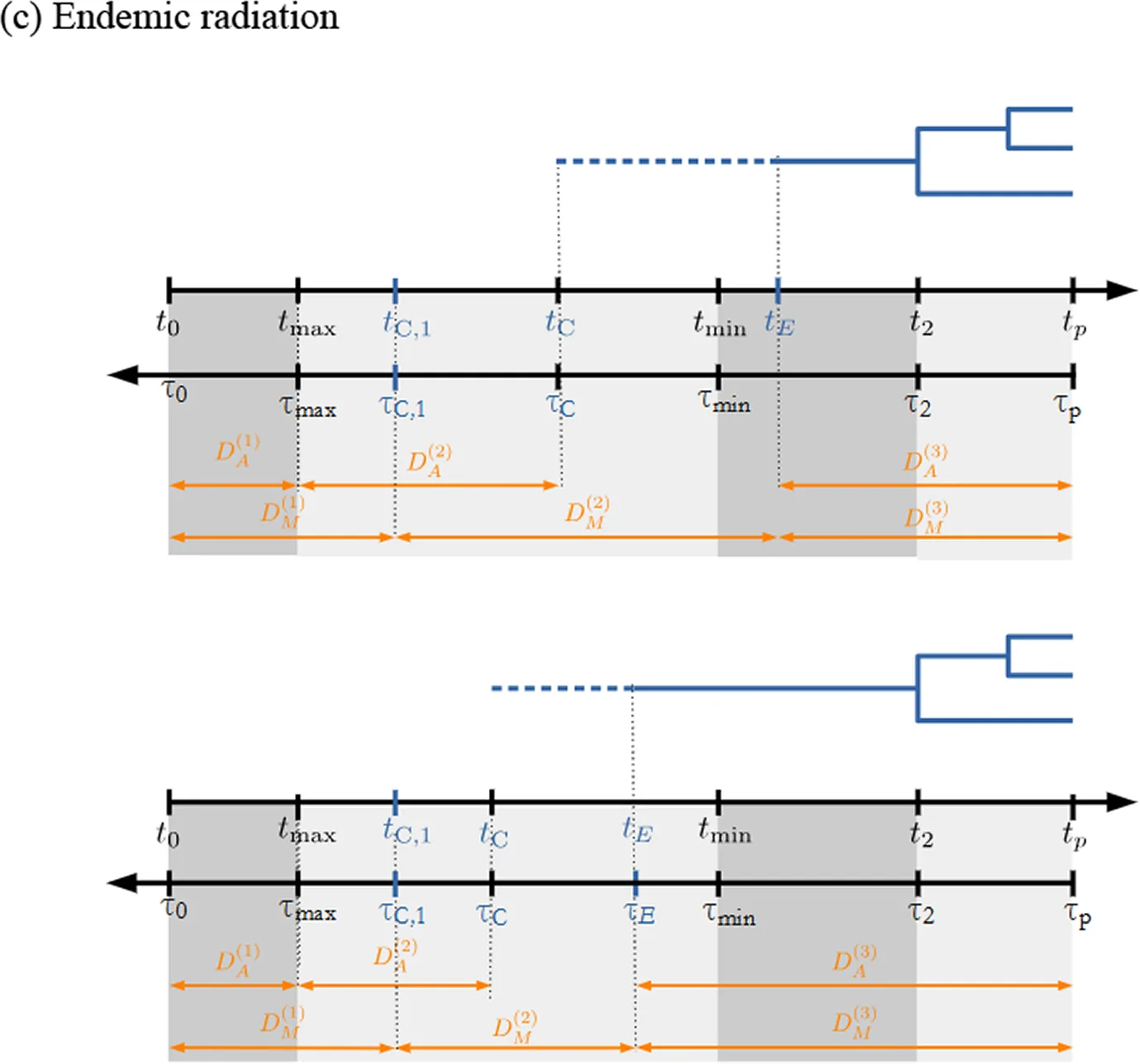
Schematic representations of the temporal structure and definition of the state probabilities $D_{\textrm{M}}({{\tau }})$ and $D_{\textrm{A}}({{\tau }})$ in the backward pruning algorithm for different cases. The blue line represents the time interval during which the species is endemic. The orange arrows show the time intervals over which each probability is computed, going backward in time. The grey-shaded regions represent different time intervals, each governed by a distinct set of differential equations. These intervals correspond to different phases in the lineage history, reflecting changes in model dynamics across time

## Appendix A Likelihood of Singleton Lineages and Lineages with Unknown Colonization Times

Here we present the likelihood calculation for singleton lineages and lineages whose estimated colonization time is unknown, and for which only an estimated maximum and/or minimum colonization time is available. Parameters and variables used in the DAISIE-DE likelihood computations are listed in Table 3, while the dynamics of all cases are summarized in Tables 4 and 5. Figure 4 provides a schematic representation of the state probabilities ${D}_{{M}}({{{\tau }}})$ and $D_{\textrm{A}}({{{\tau }}})$ defined in the model.

**Table 3:** Parameters and probability/state variables used in the DAISIE-DE likelihood computation

Symbol / term	Meaning
*Model parameters (rates)*	
$\lambda ^{c}$	Cladogenesis rate
μ	Extinction rate
$\lambda ^{a}$	Anagenesis rate
γ	Colonisation rate
*State variables (probabilities)*	
$E({{{\tau }}})$	Probability that a lineage starting at time ${\tau }$ as an endemic species leaves no descendants at the present
${D}_{\textrm{E}}({{{\tau }}})$	Probability that a lineage that started on the island at time ${\tau }$ as an endemic lineage evolves into the observed clade
$D_{\textrm{A}}^{(1)}({{{\tau }}})$	Probability that the mainland species corresponding to $D_{\textrm{M}}^{(1)}$ is absent from the island at time ${\tau }$
$D_{\textrm{A}}^{(3)}({{{\tau }}})$	Probability that the mainland species corresponding to $D_{\textrm{M}}^{(3)}$ is absent from the island at time ${\tau }$
${D}_{\textrm{M}}^{(1)}({{{\tau }}})$	Probability that the dynamics evolve into a clade consistent with the data, starting at time ${\tau }$ from an island containing a non-endemic species that is not represented in the clade; this mainland species originates from a colonisation event between $t_{0}$ and $t_{C}$, and in this state it goes extinct before the present or is replaced by the observed mainland species
${ D}_{\textrm{M}}^{(2)}({{{\tau }}})$	Probability that the dynamics result in a clade consistent with the data, starting at time ${\tau }$ from an island containing a non-endemic species that is represented in the clade; this mainland species originates from a colonisation at ${\tau }_{C}$ and can speciate to initiate the observed lineage
${D}_{\textrm{M}}^{(3)}({{\tau }})$	Probability that the dynamics lead to a clade consistent with the data, starting at time ${\tau }$ from an island containing a non-endemic species that is not represented in the clade; this mainland species originates from a colonisation after the speciation event at ${\tau }_E$ and goes extinct before the present

**Table 4:** Differential equations defined for different cases with given colonization time ${\tau }_C$ over specific time intervals. The integration runs from right to left and the initial conditions in a column connect the equations to the equations to the left. The shading colors mean that the same set of equations is being used

The times shown in black (${\tau }_0$, ${\tau }_{max}$, ${\tau }_{min}$, ${\tau }_2$ and ${\tau }_p$) are extracted from the data. In contrast, the time points shown in blue (${\tau }_{C,1}$, ${\tau }_C$ and ${\tau }_E$) are realization-dependent. These times are described as follows:

- $t_0, {\tau }_0$: time of the island’s origin, i.e. the island’s age
- $t_{max}, {\tau }_{max}$: maximum time of colonization
- $t_{min}, {\tau }_{min}$: minimum time of colonization
- $t_{C,1}, {\tau }_{C,1}$: time of first colonization event in the colonization time window $[t_{\max }, t_{\min }]$
- $t_C, {\tau }_C$: time of colonization event that gives rise the lineage observed at the present time
- $t_E, {\tau }_E$: time at which the lineage at the present time becomes endemic
- $t_p, {\tau }_p$: present day time

**Table 5:** Differential equations defined for different cases with maximum and minimum colonization times over specific time intervals. The integration runs from right to left and the initial conditions in a column connect the equations to the equations to the left. The shading colors mean that the same set of equations is being used

There are two possible orderings of these times:

- Upper panels in Figs. 4 b) and c): $t_C< t_{\min } < t_E$ (including the case $t_E = t_2$), if speciation of the observed lineages occurs after $t_{min}$
- Lower panels in Figs. 4 b) and c): $t_C< t_E < t_{\min }$, if speciation of the observed lineages occurs between $[t_\text {max},t_\text {min}]$

We construct the likelihoods by integrating a series of differential equations. To formulate these equations, it is convenient to introduce different instances of the variables $D_{\textrm{A}}$ and $D_{\textrm{M}}$:

A1a $$\begin{aligned} D_{\textrm{M}}^{(1)}({\tau })&\qquad \text {for } \tau \in [\tau _{c,1}, {\tau }_{0}] \end{aligned}$$

A1b $$\begin{aligned} D_{\textrm{M}}^{(2)}({\tau })&\qquad \text {for } {\tau } \in [{\tau }_E, {\tau }_{c,1}] \end{aligned}$$

A1c $$\begin{aligned} D_{\textrm{M}}^{(3)}({\tau })&\qquad \text {for } {\tau } \in [{\tau }_p, {\tau }_E] \end{aligned}$$

A1d $$\begin{aligned} D_{\textrm{A}}^{(1)}({\tau })&\qquad \text {for } \tau \in [{\tau }_{max}, {\tau }_{0}] \end{aligned}$$

A1e $$\begin{aligned} D_{\textrm{A}}^{(2)}({\tau })&\qquad \text {for } {\tau } \in [{\tau }_{C}, {\tau }_{\max }] \end{aligned}$$

A1f $$\begin{aligned} D_{\textrm{A}}^{(3)}({\tau })&\qquad \text {for } {\tau } \in [{\tau }_p, {\tau }_E] \end{aligned}$$

### A.1 Singleton Endemic or Non Endemic Lineage with Colonization Time at ${\tau }_C$

In this scenario, a mainland species colonizes the island at time ${\tau }_C$. For endemic singleton lineages, speciation occurs at an unknown time ${\tau }_E \in [{\tau }_C,{\tau }_p]$. Before this event, the lineage is present on the island as a non-endemic species, and its dynamics are described by $D_{\textrm{M}}^{(2)}$. After speciation, and until the present time ${\tau }_p$, the mainland ancestor may recolonize the island and subsequently go extinct again; this is captured by $D_{\textrm{M}}^{(3)}$ and $D_{\textrm{A}}^{(3)}$. For non-endemic singleton lineages, no speciation occurs, so the process remains in state $D_{\textrm{M}}^{(2)}$. The dynamical equations on $[{\tau }_p,{\tau }_C]$ are given by Eqs. (8a-8d). The equation is *E* are given by Eq. (2c)

**For endemic singleton lineages**, the initial conditions at the present time ${\tau }_p$ are given by:

A2 $$\begin{aligned} D_{\textrm{E}}({\tau }_p)\!=\!1,\quad D_{\textrm{M}}^{(2)}({\tau }_p)\!=\!0,\quad D_{\textrm{M}}^{(3)}({\tau }_p)\!=\!0,\quad D_{\textrm{A}}^{(3)}({\tau }_p)=1, \quad E({\tau }_p)=0. \end{aligned}$$

These initial conditions reflect the fact that, at the present time ${\tau }_p$, the lineage is observed as an endemic singleton.

**For non-endemic singleton lineages**, the lineage is always in the state $D_{\textrm{M}}^{(2)}({\tau })$ in the time interval $[{\tau }_p, {\tau }_C]$ because the mainland species never become endemic. The set of equation Eqs. (8a-8d) reduces to:

with initial conditions at ${\tau }_p$ given by:

A4 $$\begin{aligned} D_{\textrm{M}}^{(2)}({\tau }_p)=1,\quad E({\tau }_p)=0. \end{aligned}$$

In the interval $[{\tau }_C,{\tau }_0]$ there is no clade, and so the mainland species is not represented. Still it could have colonized the island, a possibility we describe with the variable $D_{\textrm{M}}^{(1)}$. The dynamical equations describing the change in the probabilities between ${\tau }_C$ and ${\tau }_0$ are given by Eqs. (2a-2c). The initial conditions at ${\tau }_C$ are given by:

A5 $$\begin{aligned} D_\textrm{A}^{(1)}({\tau }_C^+) \;=\; D_\textrm{M}^{(1)}({\tau }_C^+) \;=\; \gamma \,\,D_\textrm{M}^{(2)}({\tau }_C^-), \qquad {E}({\tau }_C^+) \;=\; {E}({\tau }_C^-). \end{aligned}$$

### A.2 Non-endemic Lineage with Maximum and Minimum Colonization Time

In this case, a non-endemic lineage is present on an island, with known maximum and minimum ages of colonization. In the interval $[{\tau }_{p}$, ${\tau }_{min}]$, the species is present and is non-endemic. The dynamical equations describing the change in the probabilities in this time interval are given by Eqs. (A3a-A3a). The observed species colonized the island between the maximum colonization time ${\tau }_\text {max}$, and the minimum colonization time ${\tau }_{min}$. If there is a colonization event in $[{\tau }_{0},{\tau }_\text {max}]$ and the mainland species survives until ${\tau }_\text {max}$, it should be replaced by a new colonization event after ${\tau }_\text {max}$. To impose this, we keep track in the interval $[{\tau }_{min},{\tau }_{max}]$ of whether the mainland species currently present originates from a colonization before or after ${\tau }_{max}$. Hence, we need two variables $D_{\textrm{M}}^{(1)}$ and $D_{\textrm{M}}^{(2)}$. All colonization events in $[{\tau }_{0},{\tau }_\text {max}]$ will be tracked using $D_{\textrm{M}}^{(1)}$. A new colonization after ${\tau }_{max}$ corresponds to a transition from $D_{\textrm{M}}^{(1)}$ (mainland species present, colonization before ${\tau }_{max}$) to $D_{\textrm{M}}^{(2)}$ (mainland species present, colonization after ${\tau }_{max}$). Therefore, in the interval $[{\tau }_{min}, {\tau }_{max}]$ the dynamical equations describing the change in the probabilities are given by:  with the initial conditions

In the interval $[{\tau }_{max}, {\tau }_0]$, the dynamical equations describing the change in the probabilities are given by Eqs. (2a-2c), with initial conditions

A8a $$\begin{aligned} D_{\textrm{A}}^{(1)}({\tau }_{max}^+)= &  D_{\textrm{A}}^{(2)}({\tau }_{max}^-) \end{aligned}$$

A8b $$\begin{aligned} D_{\textrm{M}}^{(1)}({\tau }_{max}^+)= &  D_{\textrm{M}}^{(1)}({\tau }_{max}^-) \end{aligned}$$

A8c $$\begin{aligned} E({\tau }_{max}^+)= &  E({\tau }_{max}^-). \end{aligned}$$

### A.3 Endemic Singleton Lineage with Maximum and Minimum Colonization Time

In this case, an endemic singleton lineage is present on an island, with known maximum and minimum ages of colonization. If there is a colonization event in $[{\tau }_{0},{\tau }_\text {max}]$ and the mainland species survives until ${\tau }_\text {max}$, it should be replaced by a new colonization event after ${\tau }_\text {max}$ (in forward time). To impose this, we should keep track in the interval $[\tau _{max},\tau _{min}]$ of whether the mainland species currently present originates from a colonization before or after $\tau _{max}$. Hence, we need two variables $D_{\textrm{M}}$ ($D_{\textrm{M}}^{(1)}$ and $D_{\textrm{M}}^{(2)}$). A new colonization after $\tau _{max}$ corresponds to a transition from $D_{\textrm{M} }^{(1)}$ (mainland species present, colonization before $\tau _{max}$) to $D_{\textrm{M}}^{(2)}$ (mainland species present, colonization after $\tau _{max}$). Between the speciation time and the present the mainland species can recolonize (and go extinct again before $\tau _p$). This possibility is described by the variables $D_{\textrm{M}}^{(3)}$ and $D_{\textrm{A}}^{(3)}$.

In the interval $[\tau _{p}$, $\tau _{min}]$, the species is present, and may either still be non-endemic or have already undergone speciation. The dynamical equations describing the change in the probabilities in this time interval are given by:  with the initial conditions at $\tau _p$ given by

A10a $$\begin{aligned} D_{\textrm{E}}(\tau _p )= &  1 \end{aligned}$$

A10b $$\begin{aligned} D_{\textrm{M}}^{(2)}(\tau _p)= &  0 \end{aligned}$$

A10c $$\begin{aligned} D_{\textrm{M}}^{(3)}(\tau _p)= &  0 \end{aligned}$$

A10d $$\begin{aligned} D_{\textrm{A}}^{(3)}(\tau _p)= &  1 \end{aligned}$$

A10e $$\begin{aligned} E(\tau _p)= &  0. \end{aligned}$$

For the interval $[\tau _{min}$, $\tau _{max}]$, the species has colonized and may or may not have undergone speciation. The dynamical equations describing the change in the probabilities in this time interval are given by:  with the initial conditions

A12a $$\begin{aligned} D_{\textrm{E}}(\tau _{min}^+)= &  D_{\textrm{E}}(\tau _{min}^-),\end{aligned}$$

A12b $$\begin{aligned} D_{\textrm{A}}^{(3)}(\tau _{min}^+)= &  D_{\textrm{A}}^{(3)}(\tau _{min}^-),\end{aligned}$$

A12c $$\begin{aligned} D_{\textrm{A}}^{(2)}(\tau _{min}^+)= &  0,\end{aligned}$$

A12d $$\begin{aligned} D_{\textrm{M}}^{(1)}(\tau _{min}^+)= &  0,\end{aligned}$$

A12e $$\begin{aligned} D_{\textrm{M}}^{(3)}(\tau _{min}^+)= &  D_{\textrm{M}}^{(3)}(\tau _{min}^-),\end{aligned}$$

A12f $$\begin{aligned} D_{\textrm{M}}^{(2)}(\tau _{min}^+)= &  D_{\textrm{M}}^{(2)}(\tau _{min}^-),\end{aligned}$$

A12g $$\begin{aligned} E(\tau _{min}^+)= &  E(\tau _{min}^-). \end{aligned}$$

For the interval $[\tau _{max}, \tau _0]$, the dynamical equations are given by Eqs. (2a-2c), with initial conditions

A13a $$\begin{aligned} D_{\textrm{A}}^{(1)}(\tau _{max}^+)= &  D_{\textrm{A}}^{(2)}(\tau _{max}^-) \end{aligned}$$

A13b $$\begin{aligned} D_{\textrm{M}}^{(1)}(\tau _{max}^+)= &  D_{\textrm{M}}^{(1)}(\tau _{max}^-) \end{aligned}$$

A13c $$\begin{aligned} E(\tau _{max}^+)= &  E(\tau _{max}^-). \end{aligned}$$

If the endemic singleton species coexists on the island with its mainland ancestors, the initial conditions of the set of equations Eqs. (A9a-A9e) are modified to

A14a $$\begin{aligned} D_{\textrm{E}}(\tau _p)= &  1 \end{aligned}$$

A14b $$\begin{aligned} D_{\textrm{M}}^{(2)}(\tau _p)= &  0 \end{aligned}$$

A14c $$\begin{aligned} D_{\textrm{M}}^{(3)}(\tau _p)= &  1 \end{aligned}$$

A14d $$\begin{aligned} D_{\textrm{A}}^{(2)}(\tau _p)= &  0 \end{aligned}$$

A14e $$\begin{aligned} D_{\textrm{A}}^{(3)}(\tau _p)= &  0 \end{aligned}$$

A14f $$\begin{aligned} E(\tau _p)= &  0. \end{aligned}$$

### A.4 Endemic Non-singleton Lineage with Maximum Colonization Time

In this case, an endemic lineage is present on an island, for which only the maximum age of colonization is known.

In the interval $[\tau _2, \tau _p]$, the species has already undergone cladogenesis speciation. For the branches represented in the clade, the dynamical equations describing the change in the probabilities in this time interval are given by Eqs. (5a-5d), with the initial conditions

A15a $$\begin{aligned} D_{\textrm{E}}(\tau _p)= &  1 \end{aligned}$$

A15b $$\begin{aligned} D_{\textrm{M}}^{(3)}(\tau _p)= &  0 \end{aligned}$$

A15c $$\begin{aligned} D_{\textrm{A}}^{(3)}(\tau _p)= &  1 \end{aligned}$$

A15d $$\begin{aligned} E(\tau _p)= &  0. \end{aligned}$$

This set of equations is integrated along the edges of the trees starting from the tips. At each branching time we have to stop the integration and set $D_{\textrm{E}}(\tau _i)$ to $\lambda ^c d\tau$ times the product of the $D_{\textrm{E}}$ functions of the merging subclades (and do not change the *E* function).

In the time interval $[\tau _{max}, \tau _{2}]$ we have to add the dynamical equation for $D_{\textrm{M}}^{(2)}$, because we know that on at least a part of the branch the species is non-endemic. The dynamical equations describing the change in the probabilities in this time interval are given by eqs. (A11a-A11g). As initial conditions for $D_{\textrm{E}}(\tau _2^+)$ we take $\lambda ^c$ (from $\lambda ^c \, \textrm{d}\tau$ which represents the probability of cladogenesis speciation over the small time interval dτ) times the product of the $D_{\textrm{E}}$ functions of the merging subclades. For $D_{\textrm{M}}^{(2)}(\tau _2^+)$ we use the same quantity multiplied by the probability $D_{\textrm{A}}(\tau _2^-)$ that the mainland species is not present on the island. This covers both cases in which the speciation event that transforms the mainland species into an endemic one occurs either between $\tau _{max}$ and $\tau _2$ or precisely at time $\tau _2$. The initial conditions are then given by:

A16a $$\begin{aligned} D_{\textrm{E}} (\tau _{2}^+)= &  \lambda ^c\,\, D_{\textrm{E}}^{(l)}(\tau _{2}^-) D_{\textrm{E}}^{(r)}(\tau _{2}^-) \end{aligned}$$

A16b $$\begin{aligned} D_{\textrm{M}}^{(1)}(\tau _{2}^+)= &  0 \end{aligned}$$

A16c $$\begin{aligned} D_{\textrm{M}}^{(2)}(\tau _{2}^+)= &  \lambda ^c\,\, D_{\textrm{E}}^{(l)}(\tau _{2}^-) D_{\textrm{E}}^{(r)} (\tau _{2}^-) D_{\textrm{A}}(\tau _{2}^-) \end{aligned}$$

A16d $$\begin{aligned} D_{\textrm{M}}^{(3)}(\tau _{2}^+)= &  D_{\textrm{M}}^{(3)}(\tau _{2}^-) \end{aligned}$$

A16e $$\begin{aligned} D_{\textrm{A}}^{(2)}(\tau _{2}^+)= &  0 \end{aligned}$$

A16f $$\begin{aligned} D_{\textrm{A}}^{(3)}(\tau _{2}^+)= &  D_{\textrm{A}}^{(3)}(\tau _{2}^-) \end{aligned}$$

A16g $$\begin{aligned} E (\tau _{2}^+)= &  E (\tau _2^-) \end{aligned}$$

If species within lineages are sampled at random (all species in the clade have the sample probability of being sampled), incomplete sampling can be accounted for by modifying the initial conditions for each tip in the tree to reflect ρ, the probability of sampling a species (following FitzJohn et al. (2009)). In this case, the initial conditions become $D_{\textrm{E}}(\tau _p) = \rho$ and $E(\tau _p) = 1 - \rho$.

In the interval $[\tau _{max}, \tau _0]$, the dynamical equations describing the change in the probabilities are given by Eqs. (2a-2c) with initial conditions

A17a $$\begin{aligned} D_{\textrm{A}}^{(1)}(\tau _{max}^+)= &  D_{\textrm{A}}^{(2)}(\tau _{max}^-),\end{aligned}$$

A17b $$\begin{aligned} D_{\textrm{M}}^{(1)}(\tau _{max}^+)= &  D_{\textrm{M}}^{(1)}(\tau _{max}^-), \end{aligned}$$

A17c $$\begin{aligned} E(\tau _{max}^+)= &  E(\tau _{max}^-). \end{aligned}$$

If the endemic singleton species coexists on the island with its mainland ancestors, the initial conditions of the set of equations Eqs. (5a-5d) are modified to:

A18a $$\begin{aligned} D_{\textrm{E}}(\tau _p)= &  1,\end{aligned}$$

A18b $$\begin{aligned} E(\tau _p)= &  0, \end{aligned}$$

A18c $$\begin{aligned} D_{\textrm{A}}^{(3)}(\tau _p)= &  0,\end{aligned}$$

A18d $$\begin{aligned} D_{\textrm{M}}^{(3)}(\tau _p)= &  1. \end{aligned}$$

## Appendix B Analytical Likelihood Expressions for Special Cases

Here we present analytical likelihood expressions for a series of special cases in order to provide additional support to both the DAISIE and DAISIE-DE models. To facilitate the derivations, we assume that both extinction and anagenesis are absent, i.e., we set $\mu = \lambda ^a = 0$. Let $t_0$ denote the island age, $t_C$ the colonization time, $t_2$ the branching time, and $t_p$ the present time. All times are expressed in forward time, so that $t_0< t_C< t_2 < t_p$.

### B.1 Dynamics Before Colonization Time

Before the colonization event of the observed lineage, the mainland species may have previously colonized the island. If this occurred, the earlier colonizing lineage must not have undergone speciation, as its descendant lineages cannot go extinct under the model assumptions. If a mainland species colonized the island prior to the estimated colonization time of the observed lineage and survive until that time, its dynamics are assumed to be taken over by that of the newly colonizing lineage, in accordance with the DAISIE and DAISIE-DE model assumptions. This gives the following dynamics before the colonization time:

B1a $$\begin{aligned} \frac{dP_0}{dt}= &  -\gamma P_0\left( t\right) \end{aligned}$$

B1b $$\begin{aligned} \frac{dP_1}{dt}= &  \gamma P_0\left( t\right) - \lambda ^c P_1\left( t\right) \end{aligned}$$

with solutions:

B2a $$\begin{aligned} P_0(t; p)= &  e^{-\gamma (t - t_0)}(1 - p\left( t\right) ) \end{aligned}$$

B2b $$\begin{aligned} P_1(t; p)= &  \frac{\gamma }{\lambda ^c - \gamma } \left( e^{-\gamma (t - t_0)} - e^{-\lambda ^c(t - t_0)}\right) (1 - p\left( t\right) ) + e^{-\lambda ^c(t - t_0)} p\left( t\right) \nonumber \\ \end{aligned}$$

where *p* is the probability that the mainland species is present at time $t_0$.

The likelihood is given by

B3 $$\begin{aligned} L_0 = P_0(t; p) + P_1(t; p) \end{aligned}$$

### B.2 Endemic Clade with Two Tips, Known Colonization Time

For an endemic clade that colonizes the island at time $t_C$ and speciated at time $t_2$, the likelihood formula is:

B4a $$\begin{aligned} L_1= &  \left( P_0(t_C; 0) + P_1(t_C; 0)\right) \gamma e^{-(\lambda ^c + \gamma )(t_2 - t_C)} \lambda ^c e^{-(2\lambda ^c + \gamma )(t_p - t_2)} \end{aligned}$$

B4b $$\begin{aligned}= &  \left( \frac{\lambda ^c}{\lambda ^c - \gamma } e^{-\gamma (t_C - t_0)} - \frac{\gamma }{\lambda ^c - \gamma } e^{-\lambda ^c(t_C - t_0)}\right) \gamma e^{-(\lambda ^c + \gamma )(t_2 - t_C)} \lambda ^c e^{-(2\lambda ^c + \gamma )(t_p - t_2)}\nonumber \\ \end{aligned}$$

The expression $\left( P_0(t_C; 0) + P_1(t_C; 0)\right)$ describes the probability dynamics before the colonization event at time $t_C$. The exponential term $e^{-(\lambda ^c + \gamma )(t_2 - t_C)}$ represents the probability of no events (neither cladogenesis, nor extinction) occurring between the colonization time $t_C$ and the cladogenesis event at $t_2$. Finally, the term $e^{-(2\lambda ^c + \gamma )(t_p - t_2)}$ accounts for the probability that both daughter lineages, resulting from cladogenesis at $t_2$ survive until the present time $t_p$ without further diversification and colonization.

### B.3 Endemic Clade with Two Tips, Unknown Colonization Time

The likelihood formula is:

B5a $$\begin{aligned} L_2= &  P_1(t_2; 0)\lambda ^c e^{-(2\lambda ^c + \gamma )(t_p - t_2)} \end{aligned}$$

B5b $$\begin{aligned}= &  \frac{\gamma }{\lambda ^c - \gamma } \left( e^{-\gamma (t_2 - t_0)} - e^{-\lambda ^c(t_2 - t_0)}\right) \lambda ^c e^{-(2\lambda ^c + \gamma )(t_p - t_2)} \end{aligned}$$

$P_1(t_2; 0)$ describes the probability of observing a singleton lineage on the island, that have colonized at a time *t* between the island age $t_0$, and the cladogenesis time $t_2$, the term $e^{-(2\lambda ^c + \gamma )(t_p - t_2)}$ accounts for the probability that both daughter lineages, resulting from cladogenesis at $t_2$ survive until the present time $t_p$ without further diversification and colonization. It can be checked that:

B6 $$\begin{aligned} L_2 = \int _{t_0}^{t_2} L_1 \, dt_C \end{aligned}$$

### B.4 Endemic Clade with Two Tips, Maximum Colonization Time

If there is a maximum colonization time $t_{m1}$, and the branching time $t_2$ is the minimum colonization time, we subtract from the probability that a colonization takes place before $t_2$ ($P_1(t_2;0))$ the probability that a colonization takes place before $t_{m1}$ without a new colonization between $t_{m1}$ and $t_2$. The likelihood formula is:

B7a $$\begin{aligned} L_3= &  \Big (P_1(t_2; 0) - P_1(t_{m1}; 0) e^{-(\lambda ^c + \gamma )(t_2 - t_{m1})} \Big ) \lambda ^c e^{-(2\lambda ^c + \gamma )(t_p - t_2)} \end{aligned}$$

B7b $$\begin{aligned}= &  \frac{\gamma }{\lambda ^c - \gamma } \Big ( e^{-\gamma (t_2 - t_0)} - e^{-\lambda ^c(t_2 - t_0)} \nonumber \\ &  - e^{-\gamma (t_{m1} - t_0)} e^{-(\lambda ^c + \gamma )(t_2 - t_{m1})} + e^{-\lambda ^c(t_{m1} - t_0)} e^{-(\lambda ^c + \gamma )(t_2 - t_{m1})} \Big ) \lambda ^c e^{-(2\lambda ^c + \gamma )(t_p - t_2)}\nonumber \\ \end{aligned}$$

$\left( P_1(t_2; 0) - P_1(t_{m1}; 0) e^{-(\lambda ^c + \gamma )(t_2 - t_{m1})} \right)$ describes the probability of observing a singleton lineage on the island, that have colonized between the time $t_{m1}$ and the cladogenesis time $t_2$. The term $e^{-(2\lambda ^c + \gamma )(t_p - t_2)}$ accounts for the probability that both daughter lineages, resulting from cladogenesis at $t_2$ survive until the present time $t_p$ without further diversification and colonization. $L_2$ can be recovered from $L_3$ by setting $t_{m1} = t_0$.

### B.5 Endemic Clade with Two Tips that Colonized at Time $t_0$

If we know that at time $t_0$ the island is already colonized by the mainland species, the likelihood would be:

B8 $$\begin{aligned} P_1(t_2; 1)\lambda ^c e^{-(2\lambda ^c + \gamma )(t_p - t_2)} = e^{-\lambda ^c(t_2 - t_0)} \lambda ^c e^{-(2\lambda ^c + \gamma )(t_p - t_2)} \end{aligned}$$

If the mainland species is present on the island with probability *p*, the likelihood is:

B9 $$\begin{aligned} L_4 =\;&P_1(t_2; p)\lambda ^c e^{-(2\lambda ^c + \gamma )(t_p - t_2)} \nonumber \\ =\;&(1 - p\left( t\right) ) \frac{\gamma }{\lambda ^c - \gamma } \left( e^{-\gamma (t_2 - t_0)} - e^{-\lambda ^c(t_2 - t_0)}\right) \lambda ^c e^{-(2\lambda ^c + \gamma )(t_p - t_2)} \nonumber \\&+ p\left( t\right) \, e^{-\lambda ^c(t_2 - t_0)} \lambda ^c e^{-(2\lambda ^c + \gamma )(t_p - t_2)} \end{aligned}$$

Here, we assume that if the mainland species is not present on the island at time $t_0$, it can immigrate immediately afterwards.

## Appendix C Relating ρ-sampling to *n*-sampling

In our approach we use the sampling fraction ρ to specify the incompleteness of an empirical phylogeny. This is referred to as ρ-sampling. By contrast, DAISIE requires the number of unsampled species to be specified, which we call *n*-sampling. For comparison of the results with our approach with DAISIE, we would like to compute the likelihood under *n*-sampling from the likelihood under ρ-sampling. The likelihoods of phylogenetic data (which we will refer to as ‘clade’) under the two types of sampling are related by:

C1 $$\begin{aligned} P_{\rho }(\text {clade})=\sum _{n=0}^{\infty } {P_n(\text {clade}) \left( {\begin{array}{c}S + n\\ n\end{array}}\right) \rho ^S (1 - \rho ) ^{n}} \end{aligned}$$

where *S* is the number of species in the clade, *n* the number of missing species, and ρ the probability of a species to be sampled and included as assumed in the ρ-sampling.

The goal here is to isolate $P_n$ from Eq. (1). To do so, we divide by $\rho ^S$ on both sides:

C2 $$\begin{aligned} \frac{P_{\rho }(\text {clade})}{\rho ^S}=\sum _{n=0}^{\infty } {P_n(\text {clade}) \left( {\begin{array}{c}S + n\\ n\end{array}}\right) (1 - \rho ) ^{n}} \end{aligned}$$

Let’s now define x=1-ρ and write the equation in terms of *x*:

C3 $$\begin{aligned} \frac{P_{1 - x}(\text {clade})}{(1 - x) ^S}=\sum _{n=0}^{\infty } {P_n(\text {clade}) \left( {\begin{array}{c}S + n\\ n\end{array}}\right) x ^{n}} \end{aligned}$$

The right-hand side is a polynomial in *x*, which we identify term by term with the Taylor expansion of the left-hand side about x=0. This yields

C4 $$\begin{aligned} P_n(\text {clade}) \left( {\begin{array}{c}S + n\\ n\end{array}}\right) = \frac{1}{n!} \frac{d^n f(x)}{dx^n} \bigg |_{x=0} \end{aligned}$$

and hence

C5 $$\begin{aligned} P_n(\text {clade}) = \frac{S!}{(S + n)!} \frac{d^n f(x)}{dx^n} \bigg |_{x=0} \end{aligned}$$

with

C6 $$\begin{aligned} f(x) = \frac{P_{1 - x}(\text {clade})}{(1 - x) ^S } \end{aligned}$$

What remains is to find a numerically stable way to determine the derivatives. Because we actually calculate the logarithm of $P_{1 - x}(\text {clade})$, it is convenient to write the *n*-th order derivative in terms of the derivatives of g(x)=logf(x):

C7 $$\begin{aligned} f^{(n)}(x) = f(x)\, B_n\!\big ( g'(x), g''(x), \dots , g^{(n)}(x) \big ) \end{aligned}$$

Here,

C8 $$\begin{aligned} g(x) = \log (f(x)) = \log (P_{1 - x}(\text {clade}) )- S \log (1 - x) \end{aligned}$$

and $B_n$ are the *n*-th order Bell polynomials, given by

C9 $$\begin{aligned} B_0 = 1, \qquad B_n = \sum _{k=1}^n \left( {\begin{array}{c}n-1\\ k-1\end{array}}\right) \, B_{n-k} \, g^{(k)}(x). \end{aligned}$$

which can be computed recursively. This is currently implemented in DAISIE.

As an alternative to the numerical evaluation of the derivatives, one can use complex function theory, and write the derivatives as contour integrals using Cauchy’s integral formula. We then have

C10 $$\begin{aligned} P_n(\text {clade}) = \frac{1}{\left( {\begin{array}{c}S+n\\ n\end{array}}\right) } \frac{1}{2\pi i} \oint \frac{f(z)}{z^{n+1}}dz \end{aligned}$$

where *f*(*z*) is the analytical continuation of *f*(*x*) defined in Eq. (C6) to the complex plane:

C11 $$\begin{aligned} f(z) = \frac{P_{1 - z}(\text {clade})}{(1-z)^S} \end{aligned}$$

and the contour goes around z=0 in the positive direction (e.g. the unit circle). This approach only works if we can evaluate $P_\rho (\text {clade})$ for complex ρ. The ODE solvers currently implemented in DAISIE do not allow this, so this will be an avenue to explore in the future.

Finally, we note that because the ODE system is linear in $D_E$, we can replace the initial condition $D_E = \rho$ by $D_E = 1$ when we at the same time replace f(z)(f(x)) by $P_{1-z}(\text {clade}) \text (P_{1-x}(\text {clade})$). Figs. 5, 6 and 7 show the comparison of the ML-estimate obtained by fitting 100 generated incomplete datasets with 50% data coverage to the DAISIE model that uses the *n*-sampling approach, and the DAISIE-DE with both the *n*- and the ρ-sampling methods. We see that the parameter estimates obtained with DAISIE-DE using *n*-sampling and with the DAISIE model are practically identical, except for the anagenesis rate. This difference may be attributed to the difficulty of estimating the anagenesis rate accurately.
